# Explainable Fuzzy Learner Modelling and Learning Analytics for Human-Centered Teacher Decision Support: A Vocational Education Case Study

**DOI:** 10.3390/e28091036

**Published:** 2026-09-20

**Authors:** Eleni Papachristou, Christos Troussas, Akrivi Krouska, Christos Papakostas, Cleo Sgouropoulou

**Affiliations:** Department of Informatics and Computer Engineering, University of West Attica, 12243 Egaleo, Greece; epapachristou@uniwa.gr (E.P.); akrouska@uniwa.gr (A.K.); cpapakostas@uniwa.gr (C.P.); csgouro@uniwa.gr (C.S.)

**Keywords:** learning analytics, Learning Record Store (LRS), Explainable Artificial Intelligence (XAI), fuzzy learner modelling, teacher decision support, vocational education

## Abstract

Artificial Intelligence (AI)-based educational systems increasingly support personalised learning, adaptive feedback, and learning analytics. However, these capabilities are often addressed separately, with comparatively less attention paid to their integration into interpretable, teacher-facing decision-support frameworks. This study presents the e-Teacher Assistant, a human-centred educational framework that integrates xAPI-style Learning Record Store (LRS) analytics, dynamic learner modelling, Sugeno-type fuzzy inference using interpretable IF–THEN rules, adaptive learning support, and a dual Student Model–Teacher Model architecture. The framework was evaluated during a three-month authentic deployment in a vocational education Computer Networks course involving 117 learners and four educators. Learner evaluation combined a structured questionnaire, open-ended responses, and LRS-based behavioural analytics, while educators evaluated the Teacher Model through a questionnaire and qualitative responses. Learners reported predominantly positive perceptions of the system, including ease of use (92.3%), usefulness of feedback (95.7%), helpful interaction with the AI Assistant (94.9%), support for independent learning (88.0%), and support for educators through learning analytics (87.2–88.0%). Fairness and objectivity were also evaluated positively by 86.3% of learners. All four educators evaluated the Teacher Model positively, and all strongly agreed on its overall usefulness and its support for progress monitoring. Prior familiarity with digital learning tools was significantly associated with evaluations across all seven questionnaire dimensions. LRS analytics further documented learner engagement, repeated assessment activity, and contextual AI use; AI-assisted examination support was recorded in 64.42% of learner–chapter records. Overall, the findings provide initial empirical support for the feasibility of integrating interpretable learner modelling, adaptive AI-assisted support, LRS-based learning analytics, and teacher-facing decision support in an authentic vocational education setting while preserving educator oversight and pedagogical responsibility.

## 1. Introduction

In recent years, Artificial Intelligence (AI) has emerged as an important driver of change in education, influencing both teaching practices and the ways in which learning processes are organised and supported. Its integration into educational settings has been associated with more personalised learning experiences, intelligent learning support systems, and automated assessment processes that can be adapted to the needs of individual learners [1]. The COVID-19 pandemic further accelerated the digital transformation of educational institutions, leading to the widespread adoption of online platforms, virtual classrooms, and digital learning tools [2]. Together, these developments have increased the demand for more flexible, personalised, and interactive learning environments capable of addressing diverse learner needs [2,3,4].

Recent advances in smart classroom technologies have further enriched digital learning environments through the integration of intelligent infrastructures and connected educational services that support interactive teaching and learning [5]. Building on these developments, AI-based educational systems have evolved to incorporate Intelligent Tutoring Systems (ITSs), educational chatbots, adaptive learning platforms, conversational agents, and learning analytics tools that support both learners and educators throughout the educational process [6,7,8,9]. Through the integration of Natural Language Processing (NLP), recommendation systems, conversational AI, and Large Language Models (LLMs), these systems can provide personalised feedback, support the monitoring of learning progress, facilitate formative assessment, and adapt educational content to individual learner needs [6,10,11,12]. Contemporary AI-based educational assistants also draw on learner analytics and interaction data to identify knowledge gaps, support pedagogical decision-making, and enhance learner engagement and participation [9,11,12,13]. In parallel, learning analytics dashboards have increasingly been used to support educators by providing actionable information about learner progress, participation, and classroom orchestration, thereby facilitating data-informed instructional decision-making [13,14]. Recent developments in emotionally responsive and conversational educational assistants have also sought to combine NLP, semantic analysis, adaptive mechanisms, and learning analytics in order to provide more personalised forms of support for both learners and educators [11].

At the same time, contemporary educational environments place increasing instructional and administrative demands on educators. Large class sizes, heavy teaching workloads, continuous assessment requirements, and administrative responsibilities may reduce the time available for sustained pedagogical support and timely intervention [15]. Although digital tools offer important opportunities for teaching and learning, their effective use often requires additional time for preparation, training, and the development of tailored educational materials. As a result, some technological solutions may create additional organisational and cognitive demands for educators rather than serving as supportive tools in everyday educational practice.

Despite substantial progress in AI-based educational assistants, many existing systems remain at the proof-of-concept stage, with limited implementation in authentic educational settings and insufficient pedagogical validation [8]. In addition, many of these systems focus primarily on responding to learners or supporting automated assessment, while offering more limited support for continuous pedagogical monitoring, interpretable evaluation of learning behaviours, and pedagogical decision-making by educators [6]. The increasing adoption of AI-powered educational systems has also intensified concerns related to transparency, data privacy, algorithmic bias, and learners’ digital well-being, particularly in learning analytics and AI-based decision-support contexts [16,17].

Against this background, the present study reports the development and implementation of an intelligent e-Teacher Assistant built using the .NET 8 framework and grounded in learner analytics, a Learning Record Store (LRS), and fuzzy logic-based learner modelling mechanisms. The proposed system incorporates dynamic learner and educator models designed to support continuous monitoring of learning activities, adaptive learning support, and data-informed pedagogical decision-making [13]. The system was implemented in authentic vocational education teaching settings, and its evaluation combined system usage data with questionnaire-based data collected from both learners and educators.

Given the need for interpretable, adaptive, and human-centred educational systems, this study was guided by the following research questions: (RQ1) How do learners perceive the usability, usefulness, and educational value of the e-Teacher Assistant, an explainable AI-based system that integrates LRS-based learning analytics with interpretable fuzzy learner modelling? (RQ2) To what extent do learners perceive the system as supporting autonomous learning, active engagement, and reflection on errors in an authentic vocational education setting? (RQ3) How do learners and educators perceive the role of the Teacher Model in supporting pedagogical monitoring and decision-making? (RQ4) How is prior familiarity with digital learning tools associated with learners’ acceptance of the system’s adaptive and explainable features?

This study makes four main contributions: (1) the design of a unified educational framework that integrates a Learning Record Store (LRS), explainable fuzzy learner modelling, and a dual Student–Teacher Model architecture within a single educational system; (2) a human-in-the-loop pedagogical decision-support mechanism in which learner analytics and interpretable fuzzy modelling contribute to system-generated learner-status labels (On Track, Monitor, and At Risk) and pedagogical recommendation flags (Needs Retheory and Needs More Practice), while allowing educators to review and override the recommended actions; (3) the deployment and evaluation of the proposed framework involving 117 learners and four educators during a three-month intervention in an authentic vocational education setting; and (4) empirical insight into how LRS-based learning analytics and explainable AI can be combined to support both personalised learning and educator-facing pedagogical decision-making while preserving the educator’s central role.

The present study represents an independent, multi-layered advancement over our prior work on algorithmic fuzzy student assessment [18], which was restricted to a preliminary pilot evaluation of a standalone assessment component. The fundamental novelty of the present work lies in: (a) architecting and deploying the full e-Teacher Assistant framework that dynamically couples an xAPI-compliant Learning Record Store (LRS) pipeline with generative AI interaction and fuzzy modeling; (b) designing and implementing a dedicated, human-in-the-loop Teacher Model providing class-level diagnostic reporting and pedagogical decision support; and (c) conducting a longitudinal, multi-perspective empirical deployment in an authentic vocational context. We explicitly confirm that there is no participant, dataset, or statistical overlap between the two studies: the empirical cohort evaluated herein (N=117 learners and n=4 educators) represents an entirely distinct cohort deployed during an independent academic period, yielding completely novel LRS interaction logs and evaluation datasets. While the core fuzzy inference equations are summarized in the Formal Specification of the Fuzzy Inference Mechanism Section to guarantee that this manuscript remains strictly self-contained, all architectural integrations, teacher-facing dashboards, behavioral LRS log analytics, and psychometric findings reported in this paper are completely original and unique to this investigation.

## 2. Related Work

### 2.1. AI in Education and Intelligent Educational Systems

In recent years, the integration of Artificial Intelligence (AI) into education has supported the development of intelligent and adaptive learning systems, including Intelligent Tutoring Systems (ITSs), Virtual Teaching Assistants (VTAs), conversational agents, and educational chatbots [14,19]. These systems use AI, Natural Language Processing (NLP), and adaptive learning techniques to provide personalised guidance, timely feedback, support for assessment activities, and improved access to learning materials, while complementing rather than replacing the pedagogical role of the educator [20,21].

The emergence of Large Language Models (LLMs), such as ChatGPT, has further increased interest in learner-centred and self-regulated learning environments, as these models can support the generation of educational content, interactive learning experiences, and feedback tailored to the needs of both learners and educators [22,23]. From a technological perspective, contemporary AI-powered educational systems rely on machine learning, deep learning, and NLP techniques to analyse educational data and learner interactions, thereby enabling more flexible and adaptive forms of learning [12,24]. In this context, conversational AI may further enhance learner interaction with the learning environment by providing timely assistance and additional opportunities for active participation [12,20].

At the same time, the educational value of AI-based systems depends not only on their technical capabilities but also on factors such as learner motivation, trust in AI virtual assistants, the quality of the feedback provided, and alignment with pedagogical principles [25,26]. Their growing adoption also raises important challenges related to transparency, response reliability, ethical data use, and the need to maintain human pedagogical oversight [22,27].

### 2.2. Explainable AI and Interpretable Learner Modelling

As AI-based educational systems are increasingly used for assessment, feedback provision, and pedagogical decision-making, the transparency and interpretability of their outputs have become increasingly important. Explainable Artificial Intelligence (XAI) is therefore widely recognised as a key prerequisite for the meaningful adoption of AI in education, particularly in contexts where system predictions influence learners’ educational pathways and assessment [27].

Despite advances in Artificial Intelligence in Education (AIED), many existing approaches still rely on black-box models, such as deep neural networks and other complex machine learning architectures, whose decision-making processes are difficult for educators and learners to interpret [27]. This limits users’ ability to understand, justify, and critically evaluate AI-generated recommendations, predictions, and assessments. The rapid integration of LLMs and generative AI into education has further reinforced the need for reliable, transparent, and human-centred approaches supported by ongoing human oversight [22,27].

In educational contexts, interpretability is especially important because feedback should not merely suggest correctness but should also support learners’ self-regulation, reflection, and active engagement [26,27]. Recent studies suggest that learners particularly value feedback that is clear, useful, supportive in tone, and pedagogically meaningful [25,26]. Accordingly, AI-generated feedback should be understandable, well justified, and adapted to the needs of learners and educators in ways that support effective interpretation and use [25,26,27,28].

To address this interpretability challenge, XAI research has proposed methods such as feature attribution techniques, Shapley values, and local explanation approaches, including LIME [27]. Although these methods can clarify the contribution of individual features to model outputs, their educational use may be limited by the difficulty of interpreting the explanations and by the need to present them in forms that are meaningful and accessible to educators and learners [27]. For this reason, more interpretable learner-modelling approaches, including fuzzy and rule-based models, have attracted increasing attention [29].

Fuzzy explainable learning analytics approaches represent learner states through linguistic variables and IF–THEN rules, using categories such as LOW, MEDIUM, and HIGH to transform learner interaction data into forms that are more understandable and pedagogically meaningful [29]. Such models can help educators interpret factors related to learning progress, participation, and the risk of failure or dropout, while also supporting longitudinal monitoring and more human-centred pedagogical decision-making [29]. In this way, interpretable learner profiles shift the emphasis from predictive accuracy alone towards a more transparent and pedagogically grounded understanding of the learning process [29].

### 2.3. Learning Analytics and Adaptive Monitoring Systems

The use of machine learning and learning analytics in education has grown considerably, particularly in applications related to monitoring learner progress, predicting learning difficulties, and supporting timely pedagogical interventions [24]. Data derived from quizzes, assessments, LMSs, and records of online participation and learner engagement can contribute to a deeper understanding of learning behaviours and learner needs [12,24].

Recent approaches extend beyond traditional academic performance prediction by incorporating learner interaction features and engagement indicators derived from Virtual Learning Environments (VLEs), LMSs, and digital learning activities, such as task participation, frequency of access to learning materials, and quiz performance [12,24,29]. These forms of analysis can support more personalised feedback, adaptive assistance, and data-informed educational decision-making [12,24].

At the same time, smart classroom monitoring approaches increasingly draw on real-time data from sensors, digital learning environments, and systems designed to capture learner interactions, behaviour, and affective indicators [29,30]. The literature suggests that the integration of deep learning, the Internet of Things (IoT), and intelligent classroom monitoring technologies may support more dynamic and adaptive forms of educational monitoring, particularly in smart classroom settings [31]. Recent work has also highlighted the value of combining behavioural, affective, and learner interaction data to provide a more comprehensive understanding of learner engagement [30]. Such multimodal evidence may support more adaptive learning processes and more effective human–AI collaboration in educational settings [32].

Within this broader context, there is growing interest in dynamic learner models that move beyond static representations of performance by monitoring learning progress over time and supporting personalised intervention, self-regulated learning, and more adaptive educational experiences [12,27,29]. However, much of the existing literature still focuses primarily on predicting academic performance or identifying learners at risk, with comparatively less attention paid to continuous pedagogical monitoring, adaptive support, and transparent learner modelling [27,29]. This highlights the need for explainable and adaptive learner-modelling frameworks that support not only prediction but also personalised learning and evidence-informed pedagogical decision-making in authentic educational settings [27,29].

### 2.4. Research Gap

Although prior research has made substantial progress in learning analytics, adaptive learning environments, and AI-based educational assistants, existing approaches often address these capabilities separately, focusing on areas such as predictive analytics, learner-facing personalisation, adaptive support, or teacher-oriented learning analytics, with comparatively less attention paid to their integration into a unified, interpretable decision-support framework for educators [12,13,24,27,29,33]. Moreover, prior work highlights the continuing challenge of supporting educators across multiple levels and teaching tasks, including real-time orchestration, learner and group monitoring, redesign, and data-informed instructional intervention in authentic educational settings [13,24].

Recent work in Explainable Artificial Intelligence (XAI) and human-centred learning analytics has highlighted the need for frameworks that go beyond prediction alone by allowing educators to understand the basis of AI-supported outputs and the recommendations or interventions generated by such systems [27,29]. In particular, interpretable rule-based models, dynamic learner modelling, and linguistic fuzzy IF–THEN representations appear promising for making learner analytics more understandable and pedagogically actionable for non-expert users [27,29]. However, prior work indicates that these capabilities are often addressed separately rather than being integrated with adaptive support, teacher-facing learning analytics, and longitudinal monitoring within a single educational framework [13,27,29].

To address this gap, this study proposes the e-Teacher Assistant, a human-centred and interpretable learner analytics framework that combines dynamic learner modelling, adaptive learning support, teacher-facing analytics, and evidence-informed support for assessment and instructional decision-making. As summarised in Table 1, many existing systems emphasise conversational AI, adaptive learner support, or explainable learner modelling in relative isolation. By contrast, the proposed e-Teacher Assistant integrates xAPI-style LRS-based analytics, interpretable fuzzy learner modelling, adaptive learner support, and class-level teacher analytics within a unified framework that was evaluated through an authentic educational deployment involving both learner and educator evaluation. Unlike our earlier pilot work [18], which operated without an LRS logging infrastructure, lacked generative AI tutoring capabilities, provided no teacher orchestration dashboard, and evaluated a different student sample, the present architecture delivers a unified, closed-loop pedagogical decision-support system tested across a 3-month continuous curriculum.

## 3. Proposed System

### 3.1. System Overview

This study presents an intelligent e-Teacher Assistant designed to support personalised learning and ongoing pedagogical monitoring in vocational education. The proposed system uses learner analytics, learning activity monitoring mechanisms, and interpretable assessment approaches to support both learners and educators throughout the learning process.

The system functions as an adaptive learning environment by collecting and using data derived from different learning activities, such as quizzes, assessments, time spent on theoretical content, progress across curriculum units, and participation indicators. These data are organised through Learning Record Store (LRS) mechanisms and are used to create dynamic learner profiles that support the provision of personalised feedback and adaptive learning recommendations.

In contrast to approaches that focus exclusively on static performance predictions or the identification of high-risk learners, the proposed system supports the continuous monitoring of learning progress at both the individual and class levels. Educators can monitor learners’ progress across curriculum units and chapters, while also accessing aggregated reports and learner analytics dashboards that support a more comprehensive understanding of the class learning profile.

Learner-status assessment is based on rule-based and fuzzy logic-based approaches, which support greater transparency and interpretability in learner modelling. By using linguistic rules and explainable learner analytics, the system is intended to provide information that educators can use when planning learning support and pedagogical interventions [18].

The design of the system follows a human-centred approach in which Artificial Intelligence functions as a support tool rather than as a mechanism for replacing the educator. Its adaptive operation is also aligned with learning support principles that emphasise guidance based on each learner’s level of understanding, learning difficulties, and individual needs.

In addition, the system design draws on adaptive learning principles associated with Vygotsky’s Zone of Proximal Development (ZPD). According to this perspective, learning progress can be supported when learners receive dynamic assistance based on their current level of understanding, learning difficulties, and individual learning needs.

Overall, the proposed e-Teacher Assistant combines learner analytics and Explainable Artificial Intelligence (XAI) with the practical needs of teaching and learning by supporting the continuous monitoring of learning trajectories, adaptive pedagogical support, and the provision of more understandable, transparent, and actionable feedback for educators.

The proposed system is based on two complementary models: a dynamic Student Model and a Teacher Model. The Student Model supports the continuous monitoring of learners’ learning trajectories and the creation of personalised learner profiles, while the Teacher Model transforms learner analytics data into pedagogically actionable reports and indicators that support the instructional process.

### 3.2. System Architecture

The architecture of the proposed e-Teacher Assistant was designed to support the continuous monitoring of learning activities, adaptive assessment, and the provision of personalised feedback in real time. The system is based on a modular, data-driven architecture that enables the collection, storage, analysis, and use of learning data derived from different educational activities and learner interactions.

The system was developed in a .NET 8 (net8.0-windows) environment using a modular software architecture and educational data management mechanisms based on a PostgreSQL database. In addition, real-time communication is supported through SignalR, enabling the immediate exchange of messages, event notifications, and interactions between learners and educators. The architecture is organised into distinct functional modules that work together to support the collection, storage, processing, and analysis of learning activities.

The system is organised around four main layers:

1. The User Interaction Layer (student–teacher interaction layer);
2. The Learning Data Collection Layer (Learning Record Store [LRS] layer);
3. The Learner Analytics and Learner Modelling Layer;
4. The Feedback and Pedagogical Support Layer.

**Figure 1:**
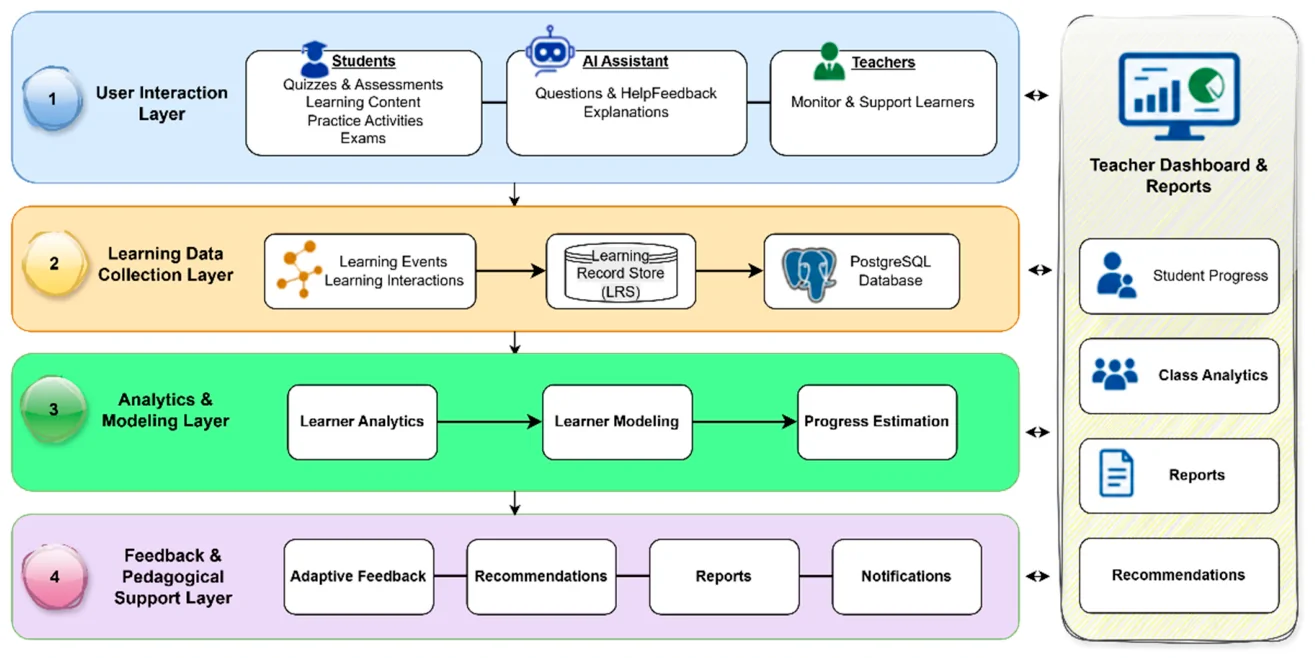
Architecture of the Proposed e-Teacher Assistant System.

The overall architecture of the proposed system is presented in Figure 1.

At the interaction layer, learners engage in various learning activities, such as quizzes, assessments, navigation through curriculum units, and interactions with educational content. Each user action is recorded as a learning event and sent to the system’s data collection mechanism. Educators interact with the system through dedicated learner analytics dashboards that provide information about learner progress, overall class performance, and the status of curriculum units.

Learning data are stored and managed through a Learning Record Store (LRS), which functions as a central repository for recording learning activities. The data are organised in a PostgreSQL database and include information such as assessment results, participation time, interaction indicators, progress across chapters, and the history of learning activities. PostgreSQL supports the efficient management of large volumes of educational data and facilitates dynamic learner analytics processes.

At the learner analytics and learner modelling layer, the system processes recorded learning data to create dynamic learner profiles for each learner. This process is based on two complementary assessment mechanisms. The first focuses on learning activity during the study of theoretical content and uses interaction, performance, and participation indicators to estimate cognitive dimensions associated with the levels of understanding, application, and analysis in Bloom’s taxonomy. The second focuses on examination performance and uses fuzzy logic-based mechanisms to estimate knowledge and skills, taking into account both learner performance and indicators of autonomy and uncertainty.

Feedback is provided through adaptive feedback mechanisms and learner analytics dashboards, which are dynamically updated based on learner profiles and assessment results. The system provides both individualised reports for learners and aggregated reports at the class level, enabling educators to identify learning difficulties, knowledge gaps, and reduced participation in a timely manner. It also supports the monitoring of performance across chapters and curriculum units, thereby facilitating more targeted pedagogical interventions.

The outputs of the individual learner-modelling mechanisms are combined into an overall learner profile that captures the learner’s learning status, level of understanding, progress across curriculum units, and indicators of participation and autonomy. This profile is used by the system’s adaptation mechanisms to generate personalised recommendations, adjust feedback, and support the learner analytics dashboards available to educators. In addition, it is used to estimate learner state and level of progress dynamically, thereby supporting the continuous monitoring of learning development [18].

The architecture was designed with transparency, scalability, and the effective use of learner analytics data in mind. In this context, the Student Model and the Teacher Model constitute the two main mechanisms for processing and pedagogically using the recorded learning data, thereby supporting both the monitoring of learning progress and educational decision-making.

#### Formal Specification of the Fuzzy Inference Mechanism

To ensure a reproducible and self-contained presentation of the interpretable assessment component embedded in the Student Model, this subsection formally specifies the fuzzy inference mechanism used to estimate learners’ cognitive states. Although the underlying mechanism builds on our previous work [18], only the components necessary for understanding its role within the overall e-Teacher Assistant framework are presented here. The examination-level learner model employs an interpretable fuzzy rule-based inference scheme that combines linguistic IF–THEN rules with fuzzy membership functions and a Sugeno-type weighted-average defuzzification strategy. This approach retains the transparency of the rule base while providing computationally efficient continuous learner-state estimates.

**Figure 2:**
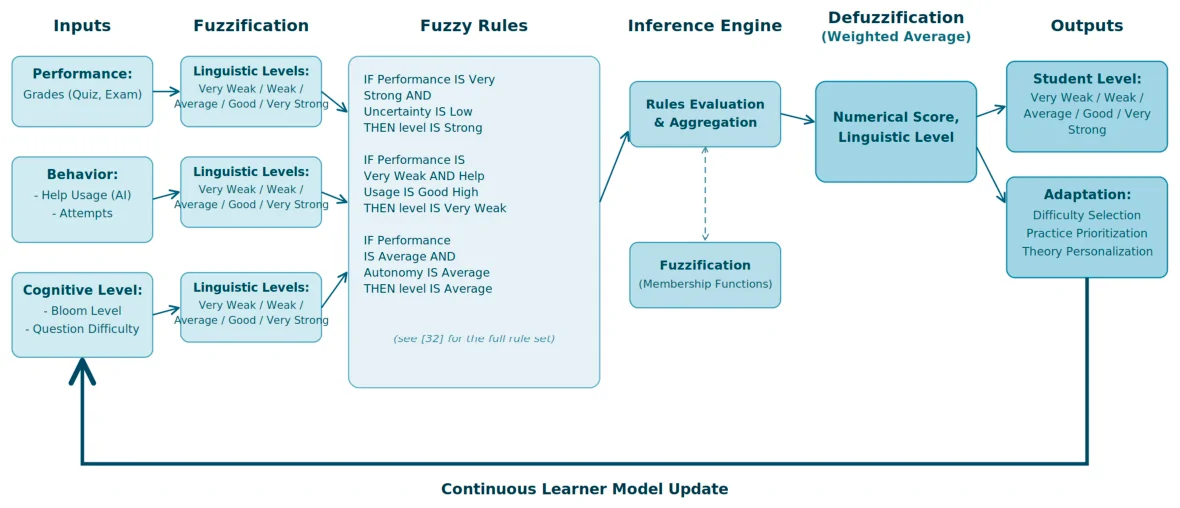
Fuzzy inference pipeline for student-level examination estimation (reconciled five-level fuzzy linguistic scale and Sugeno defuzzification, excluding formative practice activities).

The overall fuzzy inference pipeline for student-level estimation is illustrated in Figure 2.

The fuzzy inference mechanism uses three input linguistic variables. Examination Performance (P∈[0,1]) represents learners’ overall examination performance and is computed as a weighted combination of the normalised examination grade and Bloom-based cognitive evidence (Understanding, Application, and Analysis) obtained during the assessment process. When one or more assessment components are unavailable, the corresponding weights are dynamically normalised to maintain a consistent performance estimate. Autonomy ($A\in \left[0,1\right]$) and Uncertainty ($U\in \left[0,1\right]$) are operationalized strictly as computational behavioural proxies derived from real-time log telemetry rather than as psychometrically validated psychological constructs or latent cognitive traits. Within this computational framing, they capture orthogonal dimensions of help-seeking behaviour rather than duplicating the same underlying pattern. To prevent distortions arising from varying chapter lengths, Autonomy is mathematically normalized against the total number of help-eligible questions in that specific assessment, $A=1-\left(Q_{\mathrm{help}}/Q_{\mathrm{eligible}}\right)$, where $Q_{\mathrm{help}}$ is the number of distinct questions for which guidance was requested and $Q_{\mathrm{eligible}}$ is the chapter’s pool of help-enabled items. Thus, Autonomy measures the breadth (extent of independent problem attempts). In contrast, Uncertainty captures the interaction depth and conversational focus within those invoked sessions. It is formulated as

$$U=0.70\times \min\left(1.0,\frac{\bar{Turns}}{Turns_{\max}}\right)+0.30\times \left(\frac{Turns_{\mathrm{off}\text{-}\mathrm{topic}}}{Turns_{\mathrm{total}}}\right)$$

Here, $\bar{Turns}$ is the average dialogue turns per help session (normalized against an empirical threshold $Turns_{\max}=5$), and the second term penalizes unfocused dialogue. This decoupling enables the model to reward *productive help-seeking*: a learner who consults the AI for a challenging question ($Q_{\mathrm{help}}=1$), resolves the bottleneck in a concise single turn ($\bar{Turns}=1,U=0.14$), and maintains on-topic focus exhibits High Autonomy and Low Uncertainty, preserving a strong overall score. Conversely, unstructured dependency (multiple recursive turns and drifting queries) triggers High Uncertainty, appropriately moderating the learner-state estimate.

Examination Performance is represented by the linguistic categories {VERY WEAK, WEAK, AVERAGE, GOOD, VERY STRONG}, whereas Autonomy and Uncertainty are represented by the fuzzy sets {LOW, MEDIUM, HIGH}. The input universe of discourse for all three normalized antecedent variables is $x\in \left[0,1\right]$. Trapezoidal membership functions $\mu \left(x;a,b,c,d\right)$ are assigned to the extreme sets, while triangular functions $\mu \left(x;a,b,c\right)$ define intermediate sets:

- Performance (P): Very Weak $\left[0,0,0.15,0.30\right]$; Weak $\left[0.20,0.35,0.50\right]$; Average $\left[0.40,0.55,0.70\right]$; Good $\left[0.60,0.75,0.85\right]$; Very Strong $\left[0.75,0.88,1.0,1.0\right]$.
- Autonomy (A): Low $\left[0,0,0.20,0.40\right]$; Medium $\left[0.30,0.50,0.70\right]$; High $\left[0.60,0.80,1.0,1.0\right]$.
- Uncertainty (U): Low $\left[0,0,0.20,0.40\right]$; Medium $\left[0.30,0.50,0.70\right]$; High $\left[0.60,0.80,1.0,1.0\right]$.

The Sugeno zero-order defuzzification computes the crisp Exam Level Score (ELS) via the weighted average:

$$ELS=\frac{\sum_{i=1}^{M}w_{i}\cdot z_{i}}{\sum_{i=1}^{M}w_{i}}$$

Here, $w_{i}=\min\left(\mu_{P,i}\left(P\right),\mu_{A,i}\left(A\right),\mu_{U,i}\left(U\right))\right)$ denotes the firing strength of rule i, and the crisp output singletons $z_{i}$ corresponding to the five linguistic levels are: $z_{\mathrm{Very}\;\mathrm{Weak}}=0.10$, $z_{\mathrm{Weak}}=0.30$, $z_{\mathrm{Average}}=0.55$, $z_{\mathrm{Good}}=0.75$, and $z_{\mathrm{Very}\;\mathrm{Strong}}=0.95$. The continuous $ELS\in \left[0,1\right]$ is mapped to discrete linguistic categories using static interval thresholds: [0,0.25)→ Very Weak; [0.25,0.45)→ Weak; [0.45,0.65)→ Average; [0.65,0.85)→ Good; $\left[0.85,1.00\right]\to$ Very Strong.

Engagement is estimated separately from Learning Record Store (LRS) interaction data, including time spent studying theoretical content, revisits to learning material, quiz participation, and other recorded learning activities. Rather than serving as a direct input to the fuzzy inference mechanism, it is incorporated at the Overall Learner Profile stage, where it is combined with the fuzzy learner-state estimate, Bloom-based cognitive indicators, chapter-level learning analytics, concept-level learning evidence, and progression information to derive the pedagogically actionable indicators presented through the Teacher Model.

The fuzzy inference mechanism produces a continuous learner-performance estimate, referred to as the Exam Level Score (ELS∈[0,1]), which is subsequently mapped to the linguistic categories {VERY WEAK, WEAK, AVERAGE, GOOD, VERY STRONG}. The inference process is governed by a compact rule base consisting of primary performance rules and behavioural modulation rules. Examination Performance provides the initial estimate of learner proficiency, while Autonomy and Uncertainty refine this estimate by incorporating behavioural evidence related to learners’ independence during assessment and their interaction patterns during AI-assisted support. The complete rule base comprises five primary performance rules and seven behavioural modulation rules. For brevity, only representative rules are presented here, while the complete rule base, membership functions, and implementation details are described in our previous work [18].

Representative rules include:

R1: IF Performance is VERY WEAK THEN Learner Level is VERY WEAK.

R2: IF Performance is GOOD AND Autonomy is HIGH AND Uncertainty is LOW THEN Learner Level is VERY STRONG.

R3: IF Performance is AVERAGE AND Uncertainty is HIGH THEN Learner Level is WEAK.

R4: IF Performance is GOOD AND Uncertainty is HIGH THEN Learner Level is AVERAGE.

R5: IF Autonomy is LOW AND Uncertainty is HIGH THEN Learner Level is VERY WEAK.

Because the membership functions overlap, multiple rules may be activated simultaneously with different firing strengths. Each activated rule contributes to the final learner-state estimate according to its firing strength, and the final Exam Level Score is computed through a weighted-average defuzzification procedure based on predefined output centres. Consequently, rules with higher firing strengths contribute proportionally more to the final continuous learner-state estimate. This formulation produces a continuous and stable learner-performance estimate while retaining a transparent relationship between the input variables, the activated linguistic rules, and the resulting learner-state category.

The resulting Exam Level Score does not constitute the final learner assessment. Instead, it serves as one analytical component of the Overall Learner Profile, where it is integrated with chapter-level learning analytics, Bloom-based cognitive indicators, engagement metrics derived from Learning Record Store (LRS) interactions, examination and practice performance, concept-level learning evidence, help-seeking behaviour, and learner progression data. Through this integration, the system constructs a comprehensive learner profile from which two complementary types of pedagogically actionable outputs are derived: learner-status labels (On Track, Monitor, and At Risk) and recommendation flags (Needs Retheory and Needs More Practice).

These indicators are presented through the Teacher Model together with chapter-level and overall learner reports summarising cognitive performance, learning behaviour, engagement, progress trends, identified learning difficulties, and recommended instructional actions. To make the underlying fuzzy reasoning transparent and actionable during everyday instructional orchestration, the system avoids exposing raw mathematical formalisms (such as numerical membership degrees or defuzzified firing weights) directly to instructors. Instead, the explainability layer translates the activated fuzzy rules and underlying behavioral metrics into linguistically grounded, pedagogically actionable explanations. The following authentic trace illustrates how an explainable diagnostic summary is generated and presented within the educator dashboard interface:

*Learner ID:* L-1082 | *Chapter:* IPv4 Subnetting & CIDR.

*Diagnostic Assessment:* At Risk (ELS=0.400, Weak).

*Generated System Explanation:*

‘This learner scored moderately on assessment questions (raw score 10.5/20), but exhibited elevated cognitive uncertainty during problem solving. Specifically, the learner initiated 4 AI help sessions across 10 eligible items, requiring multiple conversational turns without reaching independent resolution, thereby triggering Rule R3 (Moderate Performance coupled with High Uncertainty indicates underlying foundational gaps). Although overall theory dwell time was satisfactory (48 min), formative practice accuracy remained low (52%).’

*Actionable Pedagogical Recommendation:*

‘Recommended Flags: [Needs Retheory] and [Needs More Practice]. It is suggested that the student review the conceptual boundary between host and subnet bits before attempting the examination again. The educator may confirm or dismiss this recommendation.’

This structured template explicitly links input behaviors (dwell time, score, help turns), the triggered linguistic logic (Rule R3), and the resulting instructional remediation, rendering the decision-making process auditable and interpretable for non-expert educators. Importantly, these recommendations function as decision-support mechanisms rather than automated decisions, allowing educators to review, modify, or override them where appropriate, thereby preserving human oversight and the educator’s central pedagogical role, consistent with human-centred and hybrid Human–AI approaches in education [27,32,34].

To empirically substantiate how this mathematical formulation differentiates productive assistance from cognitive dependency, and to verify that the inference engine does not penalize constructive help-seeking, a targeted sensitivity analysis was conducted.

Sensitivity Analysis of Help-Seeking Behaviour: Holding raw examination performance fixed at a representative baseline (P=0.55, raw grade 11/20), Table 2 traces the resulting Exam Level Score (ELS) and linguistic classification across four distinct behavioral archetypes under varying help-session frequencies, dialogue intensities, and off-topic ratios.

As shown in Table 2, transitioning from complete autonomy (Profile 1) to productive, targeted AI support (Profile 2) causes only a marginal score adjustment (ΔELS=−0.034), with the student comfortably maintaining their baseline linguistic category. In contrast, extensive multi-turn interactions coupled with topical deviation (Profile 4) reduce ELS by 0.290, shifting the learner from Average to Weak. This confirms that the model is selectively sensitive to behavioral inefficiency rather than penalizing help-seeking per se.

### 3.3. Student Model

The proposed system is based on a dynamic Student Model designed to support the continuous monitoring, analysis, and interpretation of learners’ learning trajectories. The Student Model functions as the core mechanism for learner analytics and adaptive learning support within the system, enabling the recording of learning activities, the creation and continuous updating of learner profiles, and the provision of personalised feedback and pedagogical support.

Unlike approaches that focus exclusively on performance assessment, the proposed Student Model is designed to support the learning process and promote learner progress. To this end, it combines data from engagement with theoretical content, practical training activities, examinations, interactions with the AI Assistant, and overall participation in the learning environment.

The model is dynamically updated using data collected through learners’ interactions with the learning environment. These data include quiz and assessment results, time spent on learning activities, frequency of interaction with the system, progress across curriculum units, the number of attempts to complete activities, and learner engagement metrics. Data are collected and stored through the Learning Record Store (LRS) mechanism, enabling the continuous updating of learner profiles and the real-time monitoring of learner progress.

**Figure 3:**
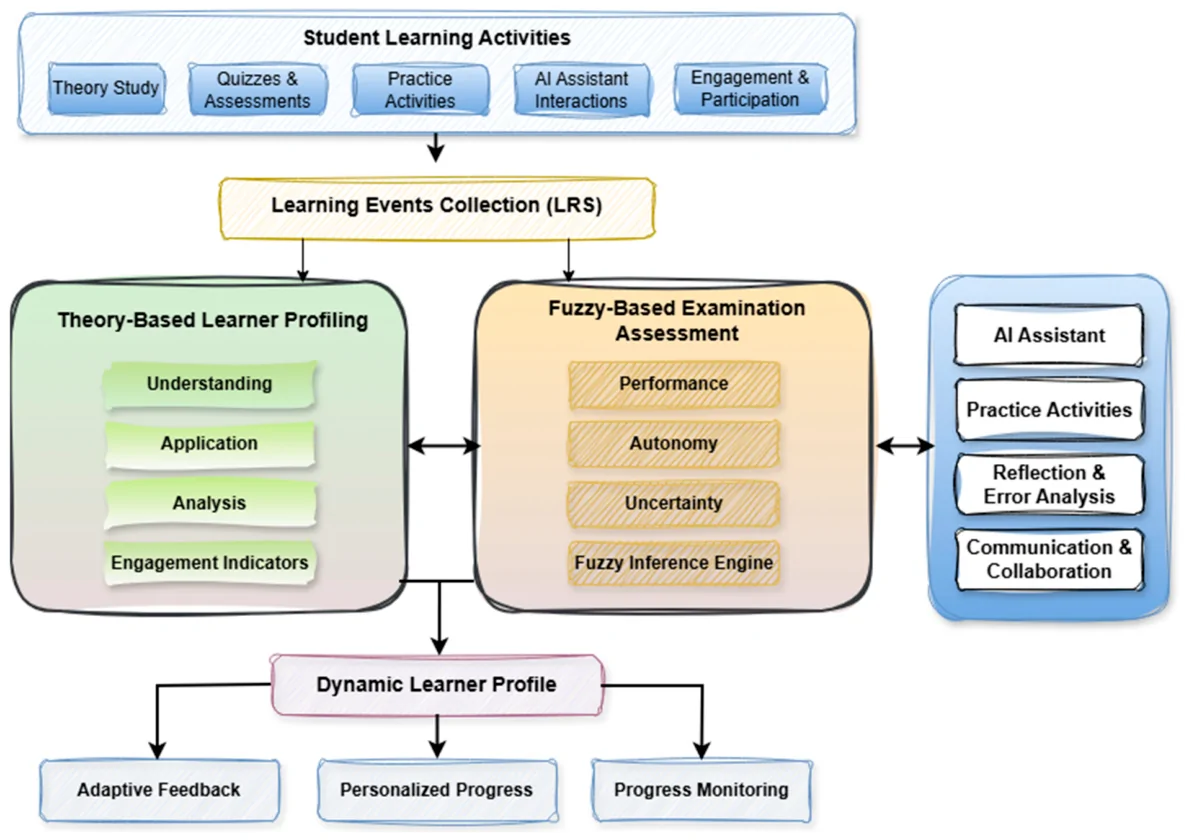
Student Model and Adaptive Learning Support Framework.

The overall structure of the proposed Student Model and its adaptive learning support mechanisms is shown in Figure 3. The model combines learner activity data, theory-based learner profiling, fuzzy-based examination assessment, and learning support mechanisms to generate a unified learner profile that supports personalised learning and the continuous monitoring of learner progress.

#### 3.3.1. Theory-Based Learner Profiling

The first level of the Student Model focuses on monitoring learning activity during engagement with theoretical content. For this purpose, the system records learning events generated through learners’ interactions with the learning material, including quiz completion, time spent studying, use of learning support mechanisms, questions addressed to the AI Assistant, and revisits to theoretical content [18].

During the learning process, learners can interact with the digital assistant to request explanations, additional examples, or clarifications regarding the concepts under study. These interactions serve as supplementary indicators of learner engagement and contribute to a more comprehensive understanding of the learning process.

These data are used to create and continuously update dynamic learner profiles that reflect learners’ level of understanding and learning progress. Assessment is based on cognitive indicators associated with Bloom’s taxonomy, particularly the levels of Understanding, Application, and Analysis. In this way, the system goes beyond the simple storage of assessment scores and provides a more comprehensive representation of learner progress and cognitive development throughout the learning process [18].

In addition, engagement indicators are calculated to support the monitoring of learners’ consistency, interaction, and overall learning behaviour. These indicators are used to assess learners’ level of active participation and may help identify possible learning difficulties or lower levels of engagement [18].

#### 3.3.2. Fuzzy-Based Examination Assessment

The second level of the Student Model focuses on assessing learners’ performance during examinations. Unlike traditional approaches that rely exclusively on the overall examination score, the proposed system uses a fuzzy logic-based assessment mechanism that supports a more interpretable representation of examination performance [18].

The assessment takes into account learners’ performance across different categories of questions and cognitive skills, as well as indicators related to autonomy and uncertainty during the problem-solving process. By using linguistic variables and IF–THEN rules, the system generates an interpretable estimate of learners’ examination status that can support the pedagogical use of the results by educators [18].

This approach provides a more detailed representation of examination performance by taking into account not only the overall examination score but also additional indicators related to the problem-solving process.

The system also distinguishes between the assessment process and the learning practice process. Practice activities are formative in nature and are not used directly to determine examination performance or learners’ final assessment. Instead, they are designed to enhance understanding and support learning improvement through repeated practice and reflection on errors.

After each examination attempt, learners are given the opportunity to review their incorrect answers, request explanations and examples from the AI Assistant, and receive additional support for concepts with which they experienced difficulties. During practice activities, the system prioritises questions related to concepts in which learners demonstrated weaknesses or gave incorrect answers in previous assessments. When no such questions are available, new activities are generated through the system’s question-generation and adaptive-content mechanisms. In this way, practice activities function as a feedback mechanism that supports the learning process by reinforcing learners’ understanding of concepts for which difficulties have been identified.

#### 3.3.3. Learning Support and Interaction Mechanisms

Beyond its monitoring and assessment mechanisms, the Student Model incorporates a set of learning support and interaction mechanisms designed to encourage learners’ active participation and support more personalised learning experiences. These mechanisms draw on learner analytics data and adapt to each learner’s needs and learning progress.

A key component of these mechanisms is the AI Assistant, orchestrated via an enterprise REST integration with the OpenAI API utilizing the gpt-4o engine (version 2024-05-13, with temperature set to 0.3 for deterministic consistency). To eliminate factual hallucination, the generation pipeline utilizes a bounded Retrieval-Augmented Generation (RAG) pattern: queries are strictly grounded against an embedded, educator-verified domain corpus covering the official vocational Computer Networks curriculum (including OSI/TCP-IP models, IPv4/IPv6 subnetting, switching, and routing protocols).

The system achieves dynamic personalization through contextual prompt augmentation. When a learner queries the assistant or requests content regeneration, the system injects the learner’s dynamic profile parameters into the system prompt:

- Cognitive Mastery Level (Bloom’s Taxonomy): Directs the response complexity (e.g., analogical and concrete foundational definitions for Remembering/Understanding vs. architectural packet-trace comparisons for Application/Analysis).
- Identified Error History: Surfaces past misconceptions recorded in the LRS to provide pre-emptive clarifications on confusing edge cases (e.g., broadcast domain vs. collision domain boundaries).
- Tone Calibration: Maintains supportive, non-punitive instructional scaffolding aligned with formative assessment goals.

Crucially, the AI Assistant operates under fundamentally different instructional constraints during study mode versus examination mode. In study mode, full theoretical expositions, step-by-step calculations, and code snippets are permitted. In examination mode, strict guardrail system prompts enforce a Socratic scaffolding protocol: the assistant is programmatically forbidden from outputting direct solutions, numerical answers, or multiple-choice key letters. Permitted guidance is restricted to: (a) clarifying domain terminology within the question stem, (b) identifying relevant conceptual principles (e.g., reminding the formula for network host calculation), and (c) providing progressive hints that guide the learner’s independent reasoning without disclosing the solution.

Because off-topic turns contribute to the Uncertainty (U) metric in the fuzzy inference engine, interaction turns are evaluated in real time via a dual filtering mechanism:

1. Semantic Similarity Gate: This computes cosine similarity between the embedded user query and the chapter’s domain ontology vectors (using text-embedding-3-small). Queries falling below an empirical similarity threshold (cosθ<0.42) trigger an initial flag.
2. Intent Classification Check: A zero-shot classification prompt classifies the flagged utterance into {Curriculum-Related, Interface/Procedural Assistance, Off-Topic/Drift}. Only conversational turns explicitly categorized as Off-Topic/Drift (e.g., chitchat, gaming, prompts attempting to jailbreak system guardrails) increment the off-topic dialogue counter ($Turns_{\mathrm{off}\text{-}\mathrm{topic}}$), ensuring that domain-adjacent or procedural help queries are never penalized.

To concretely illustrate how these prompt-conditioning mechanisms and examination guardrails operate during student learning, two contrasting authentic dialogue transcripts from the course deployment are presented below.

**Exemplar Interaction Traces:**

Example 1: Study Support Interaction (Theory Personalization).

- Context: Chapter 3: IP Addressing & Subnetting. Student profile: Level = Weak (Understanding).
- Student Prompt: ‘I don’t get how a subnet mask separates the network from the host.’
- AI Assistant Output (Profile-Conditioned): ‘Think of an IP address like a house address. The street name tells the postal worker which neighborhood you are in (Network ID), and the house number identifies your specific front door (Host ID). The subnet mask acts like a boundary fence made of 1s and 0s: wherever you see a binary 1, that part belongs to the street name; where the 0s begin, that belongs to the specific house. For example, in 255.255.255.0, the first three numbers (all 1s) lock down your network neighborhood, leaving the last number for individual computers.’

Example 2: Examination Scaffolding Interaction (Contextual Hinting).

- Context: Examination on CIDR Notation. Question: ‘How many usable host addresses are available in a /28 subnet?’
- Student Prompt: ‘What is the answer for this /28 subnet?’
- AI Assistant Output (Guardrail-Constrained Socratic Hint): ‘I cannot give you the final number directly, but let’s recall how host calculation works. An IPv4 address contains 32 bits in total. If 28 bits are reserved for the network prefix (/28), how many bits remain for host addressing? Once you find the remaining host bits (h), remember that total addresses equal $2^{h}$, and you must subtract 2 for the reserved network and broadcast addresses. Try calculating $2^{\left(32-28\right)}-2$.’

These interactions are designed to support the learning process and encourage personalised forms of guidance during independent study.

The system also supports practice activities that complement the assessment process. When generating practice activities, priority is given to concepts and questions in which learners have demonstrated difficulties or gave incorrect answers in previous attempts. In this way, practice activities are dynamically adapted to learners’ needs and are designed to support the gradual development of understanding and learning progress.

In addition, after each assessment, learners can reflect on their mistakes, review their incorrect answers, and use the system’s support mechanisms to gain a better understanding of the concepts they found challenging. This approach aligns with the principles of formative assessment by treating errors as opportunities for learning and improvement.

Finally, the system supports basic class-level communication and collaboration mechanisms, enabling learners to communicate with the educator and their peers through shared interaction channels. These features facilitate communication and the exchange of knowledge, experiences, and clarifications among class members.

#### 3.3.4. Overall Learner Profile and Adaptive Support

The Student Model brings together information derived from learning activities, assessment, and use of the system’s support mechanisms into a unified Overall Learner Profile. This profile provides an overall representation of each learner’s learning status and progress.

Specifically, it includes indicators related to:

- Overall learning progress;
- Performance across cognitive domains and at the chapter level;
- Understanding indicators based on Bloom’s taxonomy;
- Levels of participation and interaction;
- Indicators of autonomy, uncertainty, and engagement;
- An overall learner-state estimate.

The Overall Learner Profile is dynamically updated throughout the learning process, incorporating performance data together with information related to learners’ interactions, practice activities, and use of the system’s support mechanisms. As a result, it is not based exclusively on learners’ performance in assessments and examinations but is designed to provide a more comprehensive representation of learning behaviour and progress.

The continuous updating of the Overall Learner Profile supports the ongoing monitoring of learning progress and the identification of possible cognitive difficulties or lower levels of participation. The system also adopts mastery-based progression principles, according to which learners’ progress depends on their level of understanding and mastery of the curriculum units rather than solely on the time spent engaging with them.

Based on learner analytics data and the Overall Learner Profile, the system generates personalised learning recommendations, tailored feedback, and notifications for educators. These recommendations may include reviewing specific curriculum units, accessing additional learning material, engaging in alternative learning activities, undertaking targeted practice activities, and suggesting pedagogical interventions where possible learning difficulties or reduced engagement are identified.

In addition, data from the Student Model are used to generate aggregated learner analytics reports at the class level, serving as a key source of information for the Teacher Model and supporting educators in monitoring overall learner progress and informing pedagogical decision-making.

### 3.4. Teacher Model

In addition to supporting learners through the Student Model, the proposed system includes a dedicated Teacher Model designed to support monitoring, assessment, and pedagogical decision-making. The Teacher Model functions as an intermediary layer between learner analytics mechanisms and educational practice, transforming data collected from learners’ activities into information that supports educators in monitoring learner progress and informing pedagogical decision-making.

The Teacher Model uses data generated by the Student Model and learner profiles to produce personalised progress reports, aggregated performance analyses, and learner-state indicators at the learner, curriculum-unit, and class levels. Through these mechanisms, educators are provided with a more comprehensive view of learners’ learning trajectories, supporting the identification of possible cognitive difficulties, learning gaps, and reduced levels of participation.

The Teacher Model also supports continuous pedagogical monitoring by providing interpretable information about learners’ progress, curriculum units in which learners appear to experience greater difficulties, and cases in which additional educational support or intervention may be required. In this way, the system functions as a decision-support tool for educators and supports pedagogical decision-making throughout the learning process.

A distinctive feature of the proposed system is that it keeps the educator at the centre of the learning process. Although the system makes use of learner analytics, adaptive assessment mechanisms, and personalised learning recommendations, it is not designed to function as a fully automated pedagogical decision-making system. Instead, the recommendations and learner-state estimate it generates are designed to support educators, who retain final responsibility for pedagogical decisions throughout the learning process.

More specifically, learners’ progression across curriculum units is not determined solely by the system’s algorithms. Educators decide when a class is ready to move to the next curriculum unit, taking into account both learner analytics data and pedagogical considerations that cannot be fully captured by a computational model. Although the system may recommend reviewing theoretical content, engaging in additional practice activities, or following alternative learning pathways for individual learners, educators retain the ability to modify or override these recommendations where they consider this pedagogically appropriate.

This approach combines the benefits of adaptive learning and Artificial Intelligence with the central role of human pedagogical judgement, establishing a hybrid teaching framework in which technology serves as a tool to support, rather than replace, the educator.

**Figure 4:**
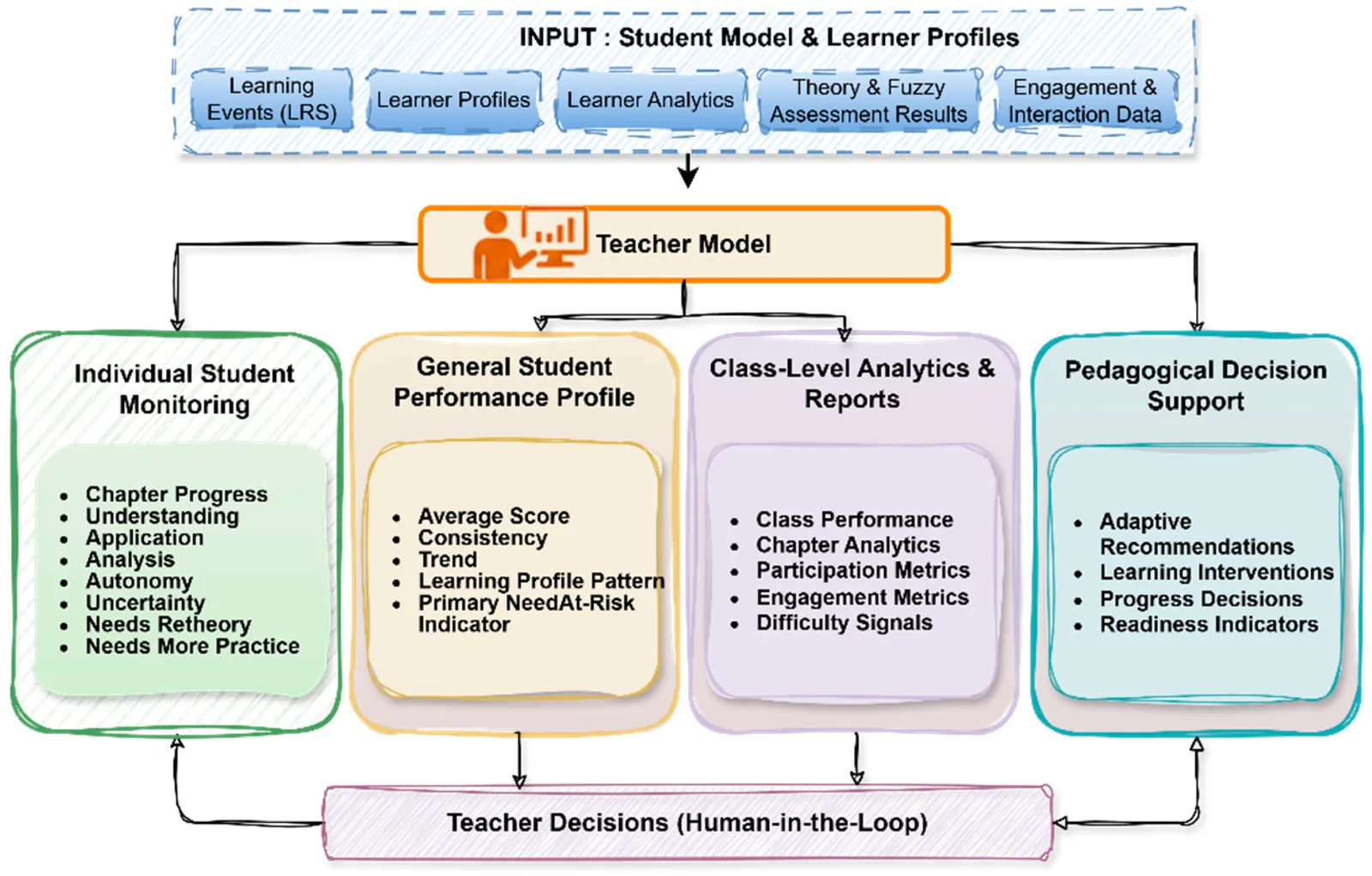
Teacher Model and Pedagogical Decision Support Framework.

The overall structure of the proposed Teacher Model is shown in Figure 4. The model integrates learner profiles, learner analytics results, and engagement indicators to support individual learner monitoring, class-level analytics, performance reporting, and pedagogical decision-making. The generated recommendations function as decision-support mechanisms, while the educator remains responsible for final pedagogical decisions.

#### 3.4.1. Individual Learner Monitoring

One of the main functions of the Teacher Model is to support the individualised monitoring of each learner’s learning trajectory. To this end, the system generates and dynamically updates reports for each learner and curriculum unit, drawing on data derived from the Student Model and the learner analytics subsystems. Monitoring is performed at the chapter level, allowing educators to examine each learner’s progress across curriculum units in greater detail.

The reports include information on learners’ progress in studying theoretical content, their performance in assessment activities, the number of attempts required to complete activities, and the overall score for each chapter.

The system draws on indicators generated by the Theory-Based Learner Profiling component to provide educators with a more detailed view of each learner’s cognitive profile.

The reports further incorporate indicators derived from the fuzzy logic-based assessment mechanism for examination activities. These include autonomy and uncertainty indicators, which provide additional information about how learners approach and manage the demands of examination activities. Together, these indicators provide educators with additional insight into learners’ learning behaviour beyond conventional score-based measures of performance.

The Teacher Model also supports the automatic identification of cases that may require additional educational intervention. In this context, the system generates indicators such as Needs Retheory and Needs More Practice, suggesting that learners may benefit from reviewing theoretical content or engaging in additional practice and preparation before the examination. These indicators are generated automatically by the system but remain under educator supervision, allowing educators to accept, modify, or override them based on their pedagogical judgement. In this way, the Teacher Model functions as a decision-support mechanism while ensuring that educators retain responsibility for final pedagogical decisions [18].

This approach combines the capabilities of Artificial Intelligence with the educator’s experience and pedagogical judgement, supporting a hybrid teaching model in which technology serves as a tool to support, rather than replace, the educator.

#### 3.4.2. General Learner Performance Profile

Beyond detailed performance monitoring at the chapter level, the Teacher Model supports the creation of an overall performance profile for each learner (General Learner Performance Profile). This profile combines data from all active chapters of the course and provides educators with an integrated view of each learner’s learning trajectory, consistency, participation, and support needs.

Through the teacher interface, educators can access the list of learners, manage their basic information, and monitor performance both at the chapter level and overall. The system presents summary information, including the overall score, general performance category, date of the most recent update, and learner progress status. In this way, educators can readily identify high-performing learners, learners who may require closer monitoring, and learners who may benefit from additional support or follow-up.

The General Learner Performance Profile includes indicators such as average score, consistency of participation, learning trend, learner profile pattern, primary learning need, and at-risk status. Rather than presenting score-based information alone, these indicators are designed to provide additional insight into the learner’s overall learning behaviour throughout the course.

The system also provides an examination history through which educators can monitor each learner’s examination attempts by chapter, including the score, completion time, and date of each attempt. This enables educators to track each learner’s progress over time.

Particular emphasis is placed on the generation of performance reports at both the learner and class levels. These reports are available both at the chapter level and for the overall course, providing educators with an integrated view of learners’ current learning status and progress. They include a concise summary of learners’ current learning status, strengths, areas for improvement, indications of low participation or learning difficulties, and suggested pedagogical actions. The system also supports the generation, export, and printing of reports at the learner, chapter, class, and overall course levels, facilitating their use in monitoring learner progress and planning targeted pedagogical interventions. In this way, learner analytics data are translated into pedagogically meaningful information that can support informed educational decision-making.

The Teacher Model further supports the generation of diagnostic reports at both the class and chapter levels. These reports summarise the overall status of the class, the quality of the available data, participation levels, the group’s cognitive profile, and the main learning difficulties identified. This enables educators to assess not only the progress of individual learners but also the overall readiness of the class to proceed to the next curriculum unit.

Overall, the General Learner Performance Profile serves as a mechanism for integrating data generated by the Student Model into an interpretable pedagogical overview. This functionality supports the educator by providing information about each learner’s progress while preserving pedagogical judgement and the ability to intervene throughout the learning process.

#### 3.4.3. Class-Level Analytics and Chapter Reports

The Teacher Model is not limited to monitoring individual learners but also supports the analysis of learning progress at the class and curriculum-unit levels. For this purpose, the system generates aggregated class-level analytics and chapter reports, enabling educators to obtain an overall view of the group’s learning status.

These reports are generated using data derived from individual learner profiles and the outputs of the learner analytics mechanisms. Through this process, indicators are calculated for overall class performance, learner participation in learning activities, and progress across curriculum units.

Particular emphasis is placed on chapter-level performance analysis. For each curriculum unit, the system calculates aggregated statistics, including average performance score, activity completion rates, learner participation, and indicators related to the cognitive dimensions of Understanding, Application, and Analysis. This analysis enables educators to identify curriculum units in which the class demonstrates greater learning difficulties or slower learning progress.

The system also calculates participation and engagement metrics that reflect learners’ level of interaction with the learning environment. These metrics enable educators to identify learners who may show lower levels of participation, reduced activity, or limited engagement with the learning material, even when assessment performance does not indicate obvious difficulties.

The Teacher Model further incorporates difficulty-detection mechanisms (difficulty signals) that draw on performance, cognitive analysis, and interaction data to indicate potentially low levels of understanding or difficulties in applying knowledge. This information is presented to educators in a way that facilitates interpretation and supports the planning of appropriate instructional interventions.

Finally, class-level reports are accompanied by automatically generated diagnostic summaries and pedagogical recommendations that summarise the main findings of the analysis and suggest possible actions for additional support, review, or reinforcement of specific cognitive skills. In this way, the Teacher Model helps translate learner analytics data into pedagogically meaningful information and supports educators in planning appropriate pedagogical interventions at the class and chapter levels.

#### 3.4.4. Pedagogical Decision Support

The main goal of the Teacher Model is not simply to present learner analytics data but to support the pedagogical process and educators’ instructional decision-making. For this purpose, the system integrates data derived from the Student Model, performance reports, and learner analytics mechanisms to provide information that helps educators plan instructional interventions and monitor learning progress.

This information is presented through personalised and aggregated reports that enable educators to identify, in a timely manner, potential learning difficulties, gaps in understanding, low levels of participation, and learners who may require additional support. The reports also provide information on progress across curriculum units, the cognitive dimensions of Bloom’s taxonomy, participation metrics, and indicators derived from the fuzzy logic-based assessment mechanism.

Specifically, status labels and recommendation flags are deterministically derived by integrating the fuzzy ELS, engagement score $E\in \left[0,1\right]$ (derived from normalized theory dwell time and mini-quiz completion), and formative practice accuracy $Acc_{\mathrm{prac}}\in \left[0,1\right]$:

- Learner Status Labels:
  ○ At Risk: ELS<0.45 OR (ELS<0.55 AND E<0.40).
  ○ Monitor: 0.45≤ELS<0.65 (with E≥0.40) OR (ELS≥0.65 AND E<0.35).
  ○ On Track: ELS≥0.65 AND E≥0.40.
- Recommendation Flags:
  ○ Needs Retheory: Triggered if ELS<0.45 OR theory engagement E<0.30, mandating theoretical subunit review.
  ○ Needs More Practice: Triggered if formative practice accuracy $Acc_{\mathrm{prac}}<0.60$ OR ELS∈[0.45,0.65), requiring completion of targeted error-remedial question pools before re-examination.

Needs Retheory indicates that the learner should return to the theoretical material before continuing, whereas Needs More Practice indicates that additional practice is required before a subsequent examination attempt. When additional practice is required, the learner must successfully complete the prescribed practice stage before access to the examination is restored. These adaptive progression rules form part of the Student Model’s learning-support mechanism rather than functioning merely as informational recommendations.

Within the Teacher Model, however, the recommendation flags remain subject to educator oversight. Educators can review and modify the Needs Retheory and Needs More Practice flags on the basis of their pedagogical judgement and knowledge of the individual learner. The learner-status labels, by contrast, represent the system’s analytical assessment of the learner’s current state and are presented to the educator as decision-support information. In this way, the system combines automated adaptive progression with human pedagogical oversight: system-generated recommendations may trigger changes in the learner’s learning pathway, but the educator can intervene in those recommended actions where pedagogically appropriate.

More broadly, the Teacher Model adopts a human-in-the-loop approach in which Artificial Intelligence functions as a collaborative support tool rather than as a substitute for the educator. This approach combines learner analytics and Artificial Intelligence with the educator’s experience, pedagogical judgement, and professional responsibility, thereby supporting pedagogical decision-making. To demonstrate the end-to-end integration of the mathematical formulations, threshold parameters, and decision logic described above, an illustrative operational trace from the authentic course deployment is presented below.

Illustrative Worked Example: Consider a learner completing Chapter 4 (Computer Networks).

1. Raw Events & Fuzzification: The student scores 10.5/20 on the examination ($P_{\mathrm{raw}}=0.525$). During the exam, they access AI help for 4 out of 10 eligible questions ($A_{\mathrm{raw}}=0.60\to$ Autonomy index A=1−0.40=0.60) and exhibit 3 extended help dialogue turns with 0 off-topic queries (U=0.35). Fuzzification yields: $\mu_{\mathrm{Average}}\left(P\right)=0.833,\mu_{\mathrm{Weak}}\left(P\right)=0.167$; $\mu_{\mathrm{Medium}}\left(A\right)=0.50,\mu_{\mathrm{High}}\left(A\right)=0.50$; $\mu_{\mathrm{Low}}\left(U\right)=0.25,\mu_{\mathrm{Medium}}\left(U\right)=0.75$.
2. Rule Firing & Defuzzification: Activating Rule R3 (IF P is Average AND U is Medium THEN Level is Weak, singleton z=0.30) with firing strength $w_{3}=\min\left(0.833,0.75\right)=0.75$, and an Average-level default rule (singleton z=0.55) with firing strength w=0.50. The defuzzified score is $ELS=\frac{\left(0.75\times 0.30\right)\;+\;\left(0.50\times 0.55\right)}{0.75\;+\;0.50}=\frac{0.500}{1.25}=0.400$ (Weak category).
3. Integration & Output Flags: The learner recorded an adequate theory study engagement (E=0.62), but achieved formative practice accuracy $Acc_{\mathrm{prac}}=0.52$. Applying the decision logic: because ELS=0.400<0.45, the system assigns the learner-status label At Risk and simultaneously activates the Needs Retheory and Needs More Practice flags. These flags lock the re-examination module and display an explainable diagnostic summary to the instructor via the Teacher Model for pedagogical review or override.

## 4. Educational Deployment

### 4.1. Context and Participants

The proposed e-Teacher Assistant was deployed during a three-month intervention in an authentic vocational education course on Computer Networks involving 117 learners and four educators. The four educators taught the participating Computer Networks classes throughout the intervention and used the Teacher Model to familiarise themselves with its functionalities, review the learning analytics it generated, and evaluate its potential to support instructional decision-making. Throughout the intervention, the system was integrated into the regular teaching process during one classroom session per week, operating as a complementary learning and pedagogical decision-support environment rather than as a replacement for conventional teaching. Learners used the adaptive learning environment to complete instructional activities, while the participating educators used the Teacher Model alongside their regular teaching practice.

For each curriculum unit, the educator first introduced the key concepts, learning objectives, and theoretical background through conventional classroom instruction. Learners then accessed the corresponding unit in the system, where they studied learning material generated dynamically according to their learner profiles.

The educational content generated by the system was tailored to each learner’s profile. Although all learners studied the same curriculum unit, the instructional content varied in level of detail, examples, explanations, and overall complexity according to each learner’s profile. In addition, learners were able to interact actively with the integrated AI Assistant by requesting clarifications, additional examples, alternative explanations, or revised versions of the theoretical material.

After completing the study of the theoretical content, learners participated in short formative quizzes designed to assess their understanding of the key concepts covered in the curriculum unit. Upon completion of all curriculum units within a chapter, learners proceeded to the practical training or examination-assessment phase, depending on the structure and learning objectives of the chapter.

After each examination phase, the educator reviewed the key concepts of the chapter and encouraged learners to reflect on their mistakes by drawing on both the system’s support mechanisms and the educator’s pedagogical guidance. Learners could use the AI Assistant to analyse incorrect answers, request additional explanations, or explore alternative approaches to solving the exercises.

Subsequently, based on learners’ performance and the indicators provided by the learner analytics system, educators encouraged learners to revisit specific curriculum units, complete additional practice activities, or participate in further assessment attempts. This process supported an iterative learning cycle that combined adaptive learning and Artificial Intelligence with the educator’s pedagogical guidance and oversight.

#### Ethics, Informed Consent, and Data Protection

The study participants comprised the full cohort of 117 adult vocational education learners (18 years of age or older) enrolled in Computer Networks courses at a public vocational education institution, together with the four educators who taught the participating classes and used the proposed Teacher Model during the three-month intervention.

All learners and educators participating in the intervention received a written information sheet explaining the purpose of the study, the types of data collected by the system (learning activity data, quiz responses, AI Assistant interaction logs, and questionnaire responses), the voluntary nature of participation, their right to withdraw at any time without academic or professional consequences, and the measures taken to ensure data confidentiality and security. Written informed consent was obtained from all participants before the start of the study.

Questionnaire responses from both learners and educators were collected anonymously, and no directly identifiable personal information was linked to the learner analytics data or the questionnaire responses used in the analysis. Personal data were processed strictly in accordance with General Data Protection Regulation (GDPR) principles. All evaluation questionnaire responses were submitted anonymously without capturing names, IP addresses, or student identifiers. System-level telemetry was assigned random alphanumeric research tokens upon account provisioning, entirely decoupling learning analytics from personal identifiers. Regarding the integration of external generative AI services, communications with the OpenAI API were routed through an enterprise endpoint operating under strict business privacy terms (zero-data retention, prohibiting data usage for model retraining). All outgoing API prompts were restricted to curriculum content, question text, and sanitized cognitive indicators, ensuring that no personally identifiable information (PII) or student credentials were ever transmitted to external AI servers.

### 4.2. Data Collection and Evaluation Procedure

The proposed system was evaluated after the completion of the three-month educational intervention. Evaluation data were collected immediately after the intervention through two electronic questionnaires administered in the classroom: one completed by learners and one by the four educators who used the Teacher Model during the intervention. A total of 117 complete learner responses were obtained, representing the entire participant cohort, together with four completed educator questionnaires. Participation in the evaluation was voluntary, and all responses were collected anonymously.

Two evaluation questionnaires were developed specifically for the present study. Their design was informed by widely used evaluation frameworks for educational technologies, including the Technology Acceptance Model (TAM) [35], the System Usability Scale (SUS) [36], the User Experience Questionnaire (UEQ) [37], established evaluation measures for Explainable AI [38], and the recent literature on XAI in educational research [39]. Both questionnaires were designed to reflect the specific functionalities of the proposed framework and the roles of learners and educators within the adaptive learning environment.

The learner questionnaire included demographic items, questions regarding prior experience with digital learning tools, and 23 evaluation statements rated on a five-point Likert scale ranging from “Strongly disagree” to “Strongly agree.” The evaluation statements were organised into seven thematic dimensions reflecting the principal functionalities and educational objectives of the proposed e-Teacher Assistant: (i) Usability and Ease of Use, (ii) Learning Support, (iii) Motivation and Persistence, (iv) Emotional Support and Interaction, (v) Perceived Usefulness, Personalisation, and Acceptance, (vi) Teacher Support and Learning Analytics, and (vii) Trust, Safety, and Human Oversight. In addition, the learner questionnaire included two open-ended questions inviting participants to identify the system’s strengths and suggest possible improvements.

The teacher questionnaire consisted of five five-point Likert-scale statements and one open-ended question designed to evaluate educators’ perceptions of the Teacher Dashboard and the Teacher Model with respect to learning analytics, learner monitoring, instructional decision support, and overall usefulness in classroom practice. The complete learner and teacher questionnaires are provided in Appendix A.

### 4.3. Data Analysis

Data analysis was conducted using IBM SPSS Statistics (Version 29.0). The analytical procedure comprised four complementary stages.

First, descriptive statistics were calculated to summarise participants’ responses and system-use data. Frequencies and percentages were used for categorical and Likert-scale questionnaire responses, while means, standard deviations, medians, and ranges were reported, where appropriate, for Learning Record Store (LRS) analytics and examination-related indicators.

For analyses conducted at the thematic-dimension level, a participant-level mean score was calculated for each of the seven learner questionnaire dimensions by averaging the Likert-scale item scores within that dimension.

Second, the internal consistency reliability of each multi-item learner questionnaire dimension was assessed using Cronbach’s alpha coefficient. Because Cronbach’s alpha is an unstable estimator for two-item scales, the Spearman–Brown coefficient was additionally computed for the two-item Motivation and Persistence dimension, as recommended for scales of this length [40]. A value of α≥0.70 was considered indicative of acceptable internal consistency, while coefficients below this threshold were interpreted with greater caution.

Third, inferential analyses were conducted to examine associations among the questionnaire dimensions and differences according to learners’ prior familiarity with digital learning tools. Spearman’s rank-order correlation coefficient (ρ) was used to examine associations among the seven-dimension scores.

For group comparisons, participants were classified according to their prior use of digital learning tools into three groups: rare or non-users (n=9), occasional users (n=32), and frequent or very frequent users (n=76). Kruskal–Wallis H tests were used to examine differences among the three independent groups across the seven questionnaire dimensions. Where statistically significant omnibus differences were identified, pairwise Mann–Whitney U tests were conducted as post hoc comparisons. The multiple-comparison family was defined at the dimension level, applying a Bonferroni-adjusted alpha threshold of $\alpha_{\mathrm{adjusted}}=0.05/3=0.0167$ to control the family-wise error rate across the three pairwise contrasts per construct. Because Likert-scale responses inherently generate tied ranks, standard asymptotic tests can yield biased variance estimates; furthermore, the rare/non-user subgroup is small (n=9). To ensure exact inference without relying on large-sample asymptotic assumptions, all pairwise Mann–Whitney U tests were calculated using exact permutation tests with standard corrections for ties. Effect sizes for pairwise contrasts were calculated using the rank-biserial correlation $r=1-2U/\left(n_{1}n_{2}\right)$ accompanied by 95% confidence intervals (CIs) estimated via bootstrapping (1000 resamples), where absolute values of 0.10, 0.30, and 0.50 denote small, medium, and large effect sizes, respectively. For the omnibus Kruskal–Wallis tests, 95% CIs for $\eta_{H}^{2}$ were likewise calculated using bootstrap estimation. Effect sizes for the omnibus Kruskal–Wallis tests were estimated using eta-squared based on the H statistic ($\eta_{H}^{2}$), calculated as $\eta_{H}^{2}=\left(H-k+1\right)/\left(N-k\right)$, where H is the test statistic, k=3 is the number of groups, and N=117 [41]. For the remaining inferential analyses, statistical significance was evaluated at p<0.05 using two-tailed tests.

Fourth, qualitative responses to the open-ended questionnaire items were analysed. Learner responses to the two open-ended questions were examined using reflexive thematic analysis following Braun and Clarke’s six-phase procedure [42]. To ensure analytic rigor, two researchers independently coded the raw qualitative corpus following initial familiarization. Initial descriptive codes were developed inductively and compared across coders, demonstrating high inter-coder agreement (Cohen’s κ=0.84). Disagreements or borderline classifications were discussed and resolved through consensus meetings involving a third senior researcher. Non-informative responses, such as punctuation-only entries or blank submissions, were excluded prior to theme synthesis. Candidate themes were iteratively grouped, cross-checked against the underlying text segments, and refined into final thematic categories representing recurring dimensions of learner experience. The open-ended responses provided by the four participating educators were examined qualitatively to identify perceived strengths of the Teacher Model and suggestions for future improvement.

Finally, regarding Learning Record Store (LRS) telemetry, the dataset contained 163 learner–chapter records generated by 117 unique learners, meaning a subset of participants contributed multiple observations across different instructional units. To address potential non-independence and within-subject clustering, engagement and examination indicators were calculated at the unit record level to reflect chapter-specific workload, and secondary verification was conducted by computing participant-aggregated mean metrics across learners’ active units; these aggregated checks yielded essentially identical distributions and identical statistical conclusions, confirming that within-subject clustering did not distort the reported descriptive benchmarks.

## 5. Results

### 5.1. Participant Characteristics

A total of 117 vocational education learners participated in the evaluation of the proposed e-Teacher Assistant system. All participants used the system during a three-month educational intervention in a Computer Networks course.

**Figure 5:**
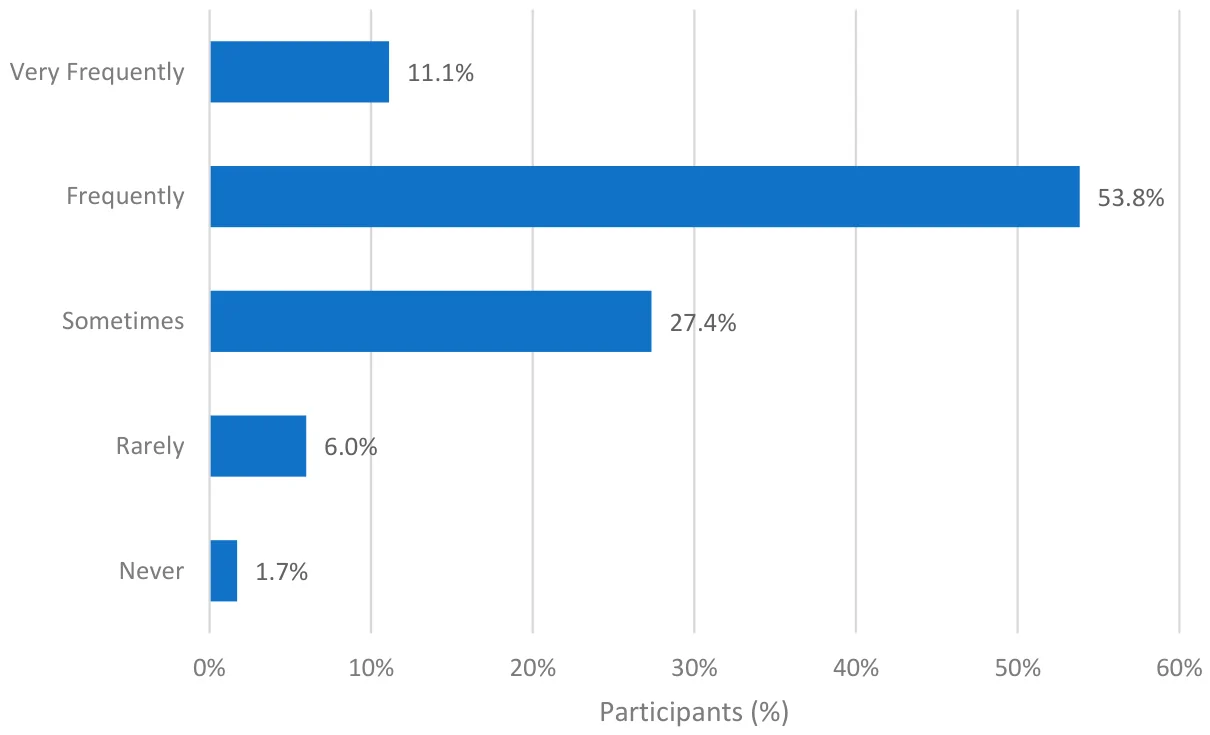
Frequency of Digital Learning Tool Usage among Participants.

The distribution of prior digital learning tool use is shown in Figure 5.

Participants reported varying levels of prior experience with digital learning tools. More than half of the participants (53.8%) indicated frequent use of digital learning tools, while 27.4% reported occasional use. Smaller proportions reported very frequent use (11.1%), rare use (6.0%), and no prior use (1.7%).

### 5.2. Usability and Ease of Use

**Figure 6:**
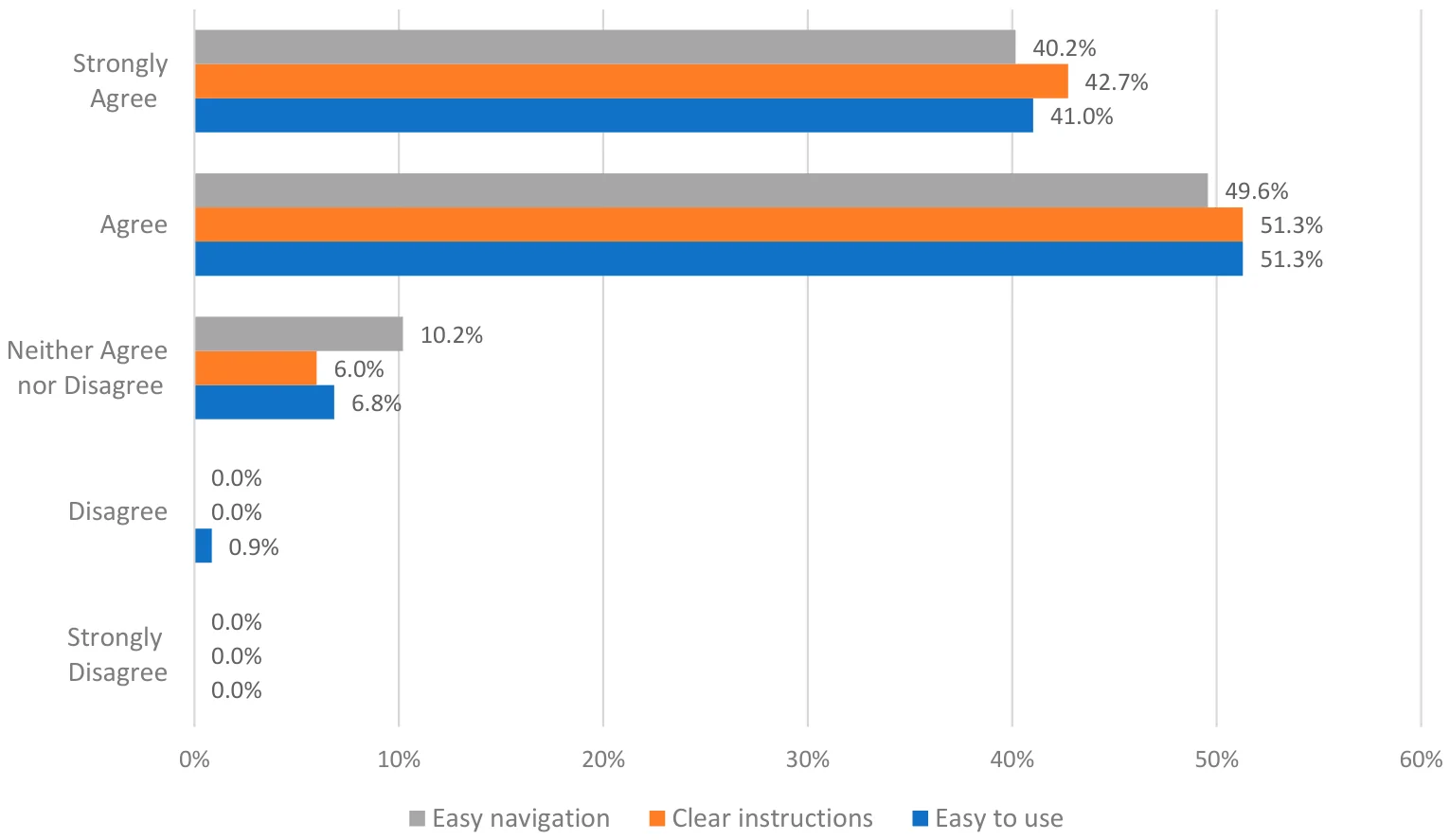
Participants’ evaluation of the usability and ease of use of the e-Teacher Assistant.

Usability was assessed in terms of overall ease of use, clarity of instructions, and ease of navigation. Responses were predominantly positive across all three items (Figure 6). Overall, 92.3% of participants agreed or strongly agreed that the application was easy to use, while 94.0% evaluated the clarity of the instructions positively and 89.7% did so for ease of navigation.

For overall ease of use, 51.3% of participants selected Agree and 41.0% selected Strongly Agree, while 6.8% provided a neutral response and 0.9% disagreed. Regarding instruction clarity, 51.3% selected Agree and 42.7% selected Strongly Agree, while 6.0% provided a neutral response. For ease of navigation, 49.6% selected Agree and 40.2% selected Strongly Agree, while 10.3% selected a neutral response. No disagreement was recorded for either instruction clarity or ease of navigation.

### 5.3. Perceived Usefulness and Personalization

#### 5.3.1. Learning Support

The second dimension examined participants’ perceptions of the learning support provided by the e-Teacher Assistant, focusing on improved understanding of the course material, identification of mistakes, usefulness of feedback, and perceived benefits of feedback.

**Figure 7:**
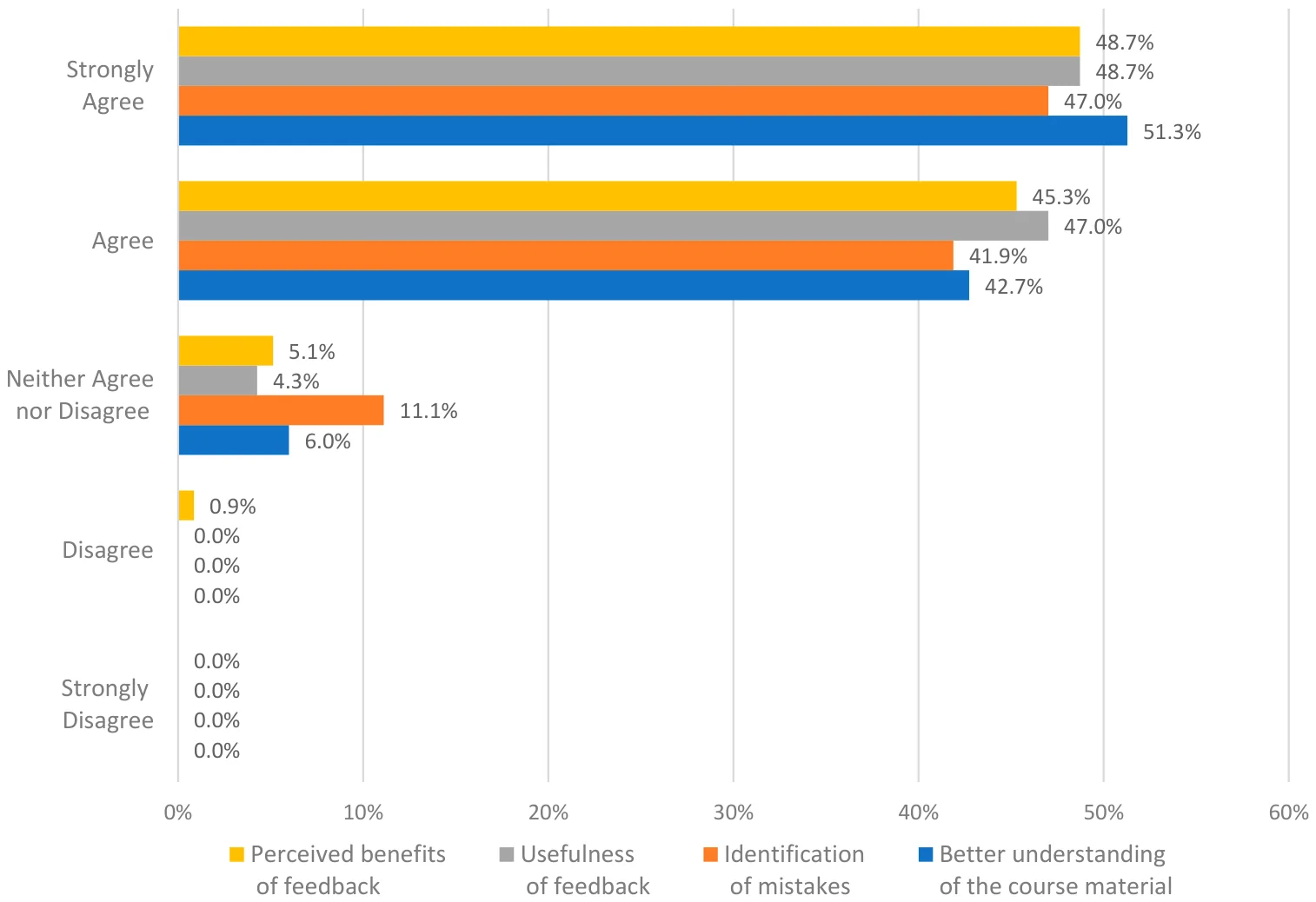
Participants’ perceptions of feedback usefulness and learning support provided by the e-Teacher Assistant.

Responses were predominantly positive across all four items (Figure 7). For improved understanding of the course material, 94.0% of participants selected Agree or Strongly Agree, while 6.0% provided a neutral response. Regarding the identification of mistakes, 88.9% of participants selected Agree or Strongly Agree, while 11.1% provided a neutral response. For the usefulness of feedback, 95.7% of participants selected Agree or Strongly Agree, while 4.3% provided a neutral response. Similarly, 94.0% of participants selected Agree or Strongly Agree regarding the perceived benefits of feedback, while 5.1% provided a neutral response and 0.9% selected Disagree. No Strongly Disagree responses were recorded for any of the four items.

#### 5.3.2. Motivation and Persistence

This evaluation dimension examined participants’ perceptions of comfort with making mistakes and the extent to which the e-Teacher Assistant encouraged them to learn from their mistakes.

**Figure 8:**
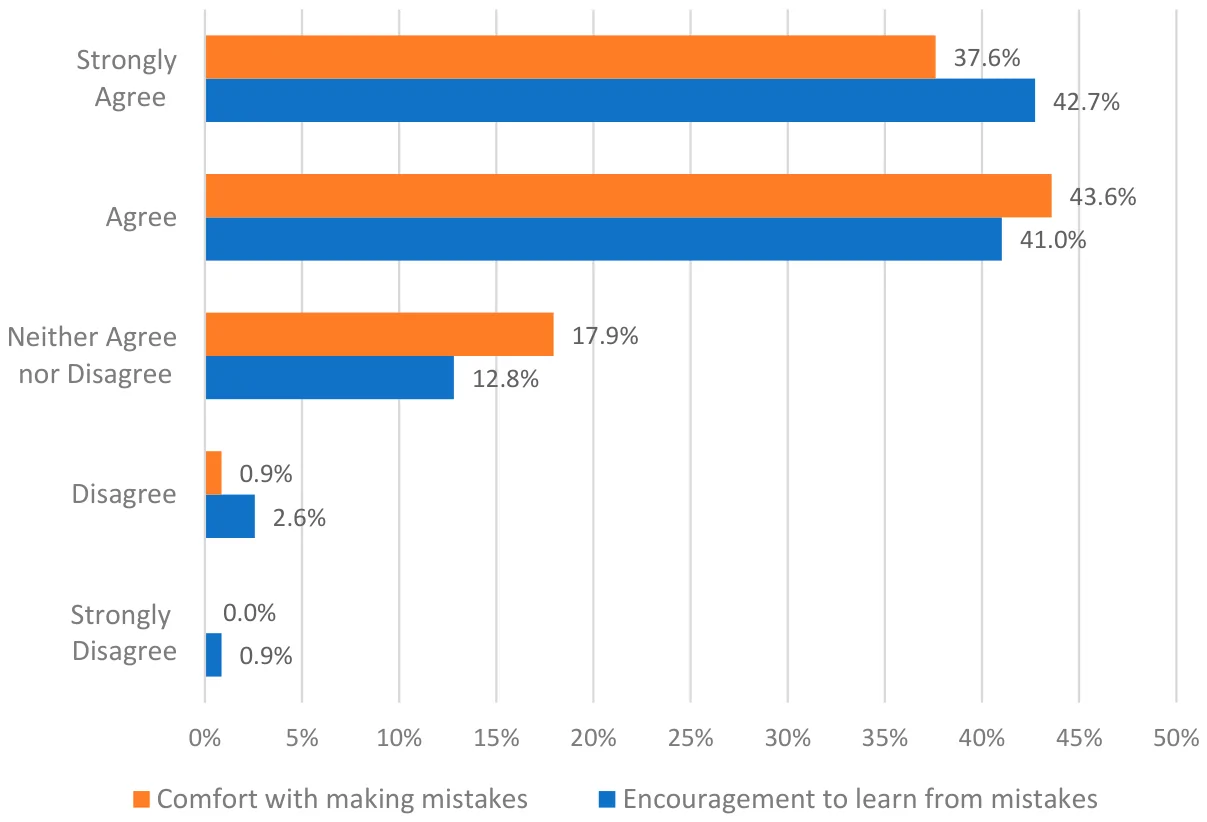
Participants’ evaluation of motivation and persistence.

As shown in Figure 8, 81.2% of participants agreed or strongly agreed that they felt comfortable making mistakes, with 43.6% selecting Agree and 37.6% selecting Strongly Agree. A further 17.9% provided a neutral response, while 0.9% selected Disagree. No participants selected Strongly Disagree.

Regarding encouragement to learn from mistakes, 83.8% of participants selected Agree or Strongly Agree, with 41.0% selecting Agree and 42.7% selecting Strongly Agree. Neutral responses accounted for 12.8%, while 2.6% selected Disagree and 0.9% selected Strongly Disagree.

#### 5.3.3. Emotional Support and Interaction

This dimension examined participants’ perceptions of the supportive role of the e-Teacher Assistant, particularly in relation to learning from mistakes, persistence after incorrect responses, and interaction with the assistant.

**Figure 9:**
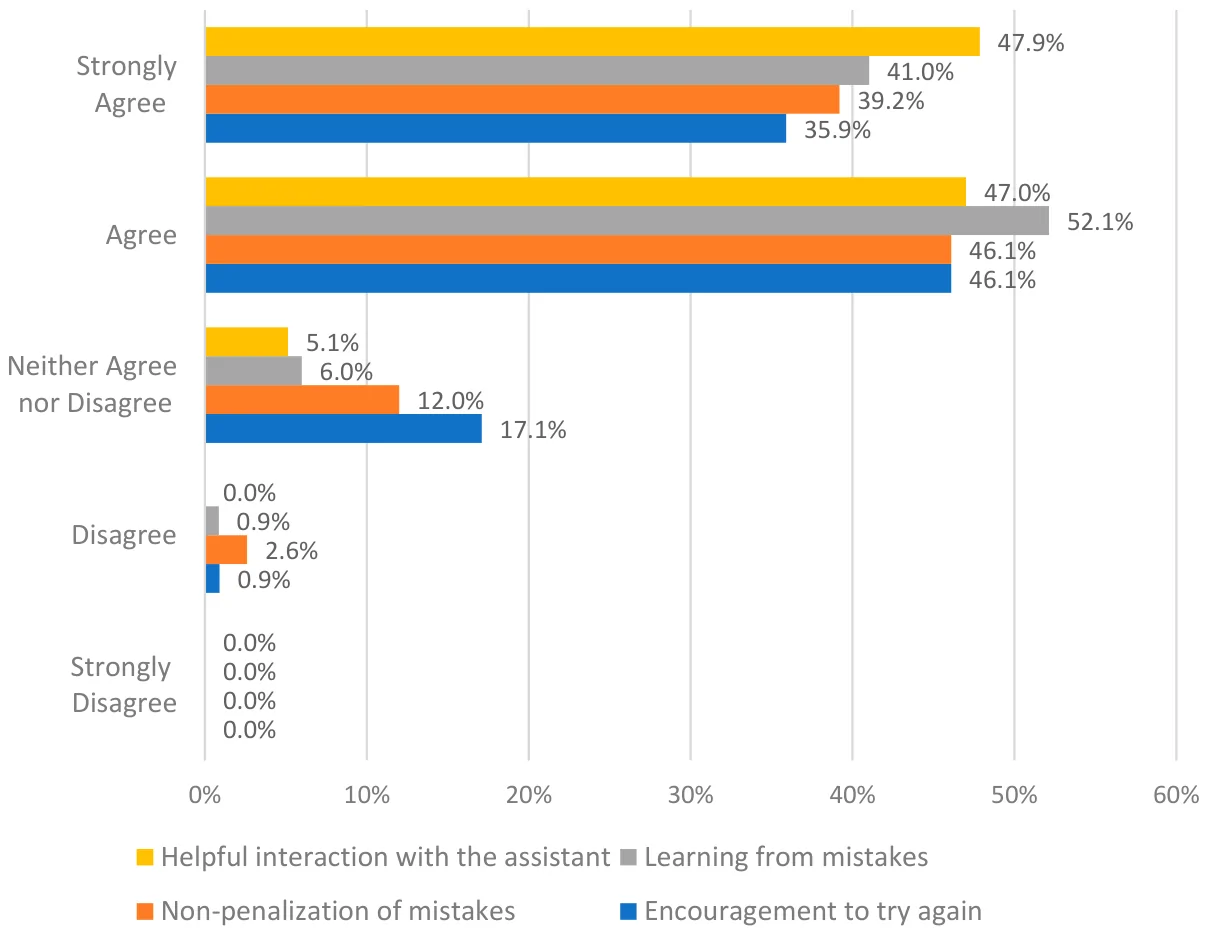
Participants’ perceptions of emotional support and interaction with the e-Teacher Assistant.

As shown in Figure 9, responses were predominantly positive across all four items. Helpful interaction with the assistant received positive responses from 94.9% of participants, with 47.0% selecting Agree and 47.9% selecting Strongly Agree. The remaining 5.1% provided a neutral response, with no disagreement recorded.

Regarding learning from mistakes, 93.2% of participants selected Agree or Strongly Agree, while 6.0% provided a neutral response and 0.9% selected Disagree.

For non-penalisation of mistakes, 85.3% of participants selected Agree or Strongly Agree. Neutral responses accounted for 12.0%, while 2.6% selected Disagree.

Finally, 82.1% of participants agreed or strongly agreed that the system encouraged them to try again, with 46.2% selecting Agree and 35.9% selecting Strongly Agree. A further 17.1% provided a neutral response, while 0.9% selected Disagree. No participants selected Strongly Disagree.

#### 5.3.4. Perceived Usefulness, Personalisation and Acceptance

This evaluation dimension examined learners’ perceptions of the e-Teacher Assistant in terms of adaptation to learning needs, level of challenge, support for independent learning, and willingness to use a similar application in other courses.

**Figure 10:**
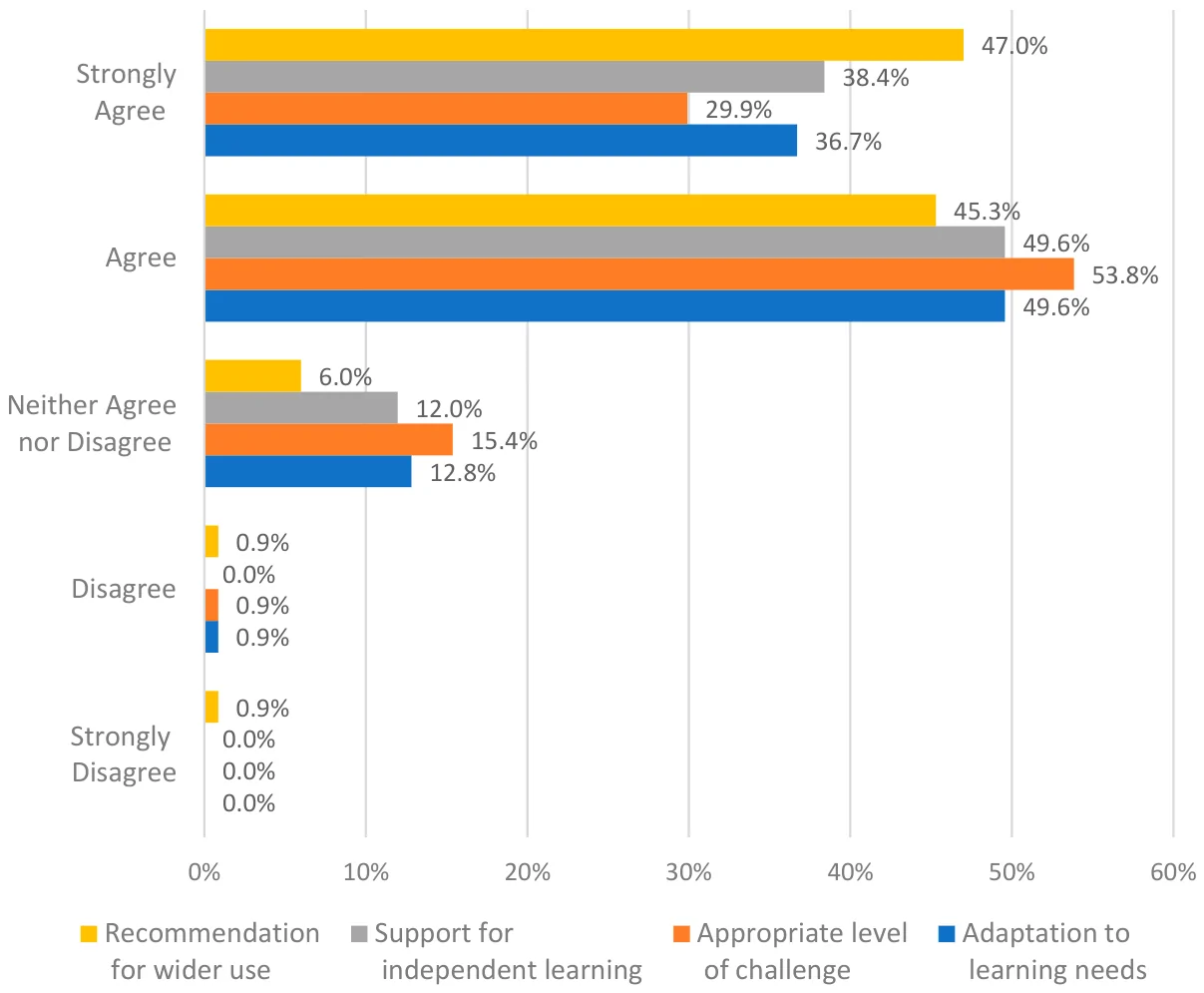
Perceived usefulness, personalisation, and learner acceptance of the e-Teacher Assistant.

As shown in Figure 10, responses were predominantly positive across all four aspects. The highest proportion of positive responses was recorded for willingness to recommend wider use of the system, with 92.3% of participants selecting Agree or Strongly Agree. Specifically, 45.3% selected Agree and 47.0% selected Strongly Agree, while 6.0% provided a neutral response. A total of 1.7% selected Disagree or Strongly Disagree.

Support for independent learning was positively evaluated by 88.0% of participants, with 49.6% selecting Agree and 38.5% selecting Strongly Agree. Neutral responses accounted for 12.0%, and no disagreement was recorded.

Regarding the appropriate level of challenge, 83.8% of participants selected Agree or Strongly Agree, comprising 53.8% and 29.9%, respectively. A further 15.4% provided a neutral response, while 0.9% selected Disagree.

Finally, 86.3% of participants responded positively regarding adaptation to learning needs, with 49.6% selecting Agree and 36.8% selecting Strongly Agree. Neutral responses accounted for 12.8%, while 0.9% selected Disagree. No Strongly Disagree responses were recorded for this item.

### 5.4. Teacher Support and Learning Analytics

This evaluation dimension examined learners’ perceptions of the contribution of the e-Teacher Assistant to teacher support through learning analytics. Specifically, participants evaluated whether the system helped educators identify individual and class-level learning difficulties and adapt instruction to learners’ needs.

**Figure 11:**
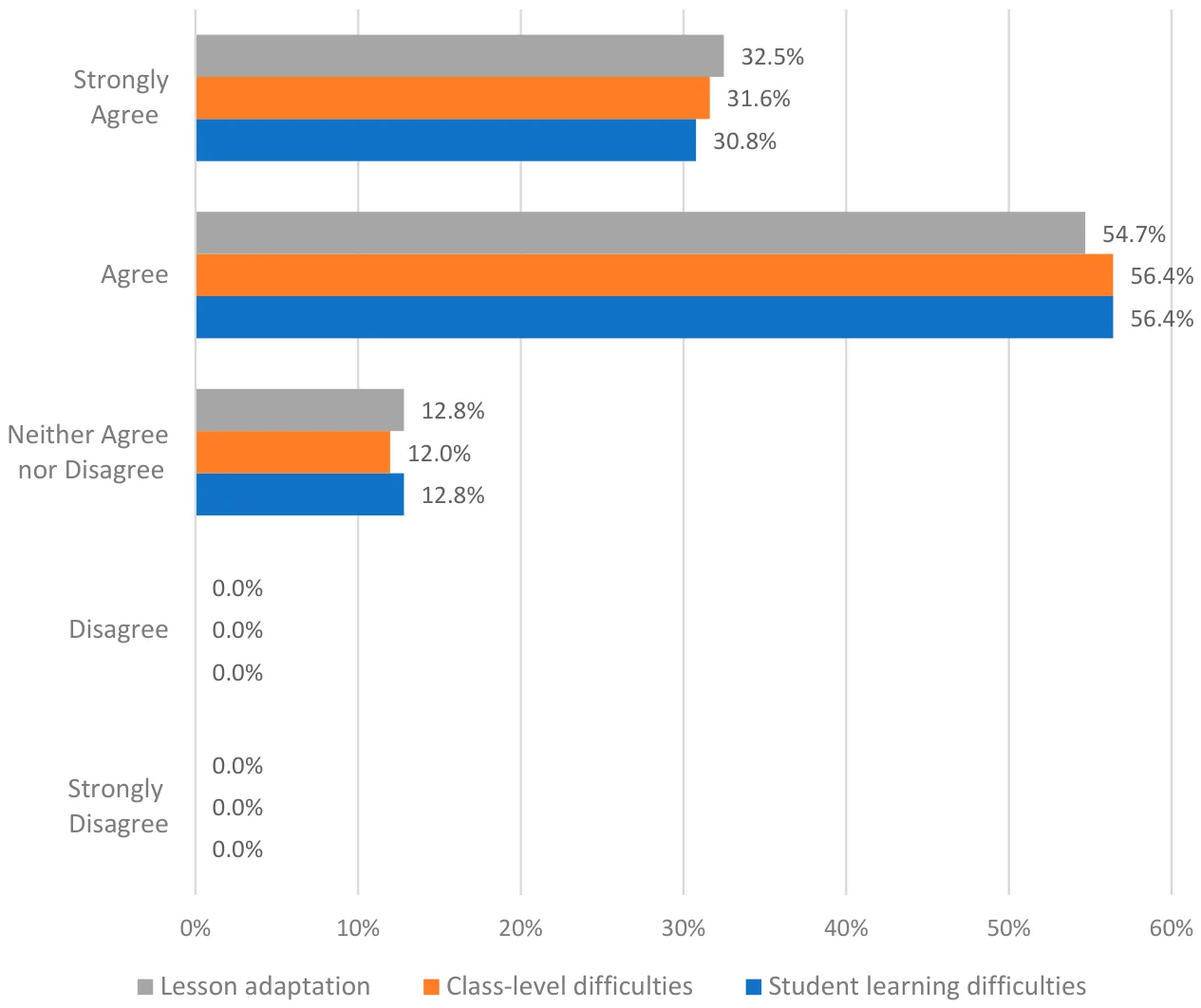
Learners’ perceptions of teacher support and learning analytics provided by the e-Teacher Assistant.

As shown in Figure 11, responses were predominantly positive across all three aspects. Regarding the identification of individual learners’ learning difficulties, 87.2% of participants selected Agree or Strongly Agree, with 56.4% selecting Agree and 30.8% selecting Strongly Agree. The remaining 12.8% provided a neutral response.

For the identification of class-level difficulties, 88.0% of participants selected Agree or Strongly Agree, comprising 56.4% and 31.6%, respectively. A further 12.0% provided a neutral response.

Regarding lesson adaptation, 87.2% of participants selected Agree or Strongly Agree, with 54.7% selecting Agree and 32.5% selecting Strongly Agree. Neutral responses accounted for 12.8%.

No participants selected Disagree or Strongly Disagree for any of the three items.

### 5.5. Trust, Safety, and Human Oversight

This evaluation dimension examined learners’ trust in the e-Teacher Assistant, focusing on the perceived fairness and objectivity of its responses, learners’ sense of safety when using the system, and their preference for verifying its responses with the educator.

**Figure 12:**
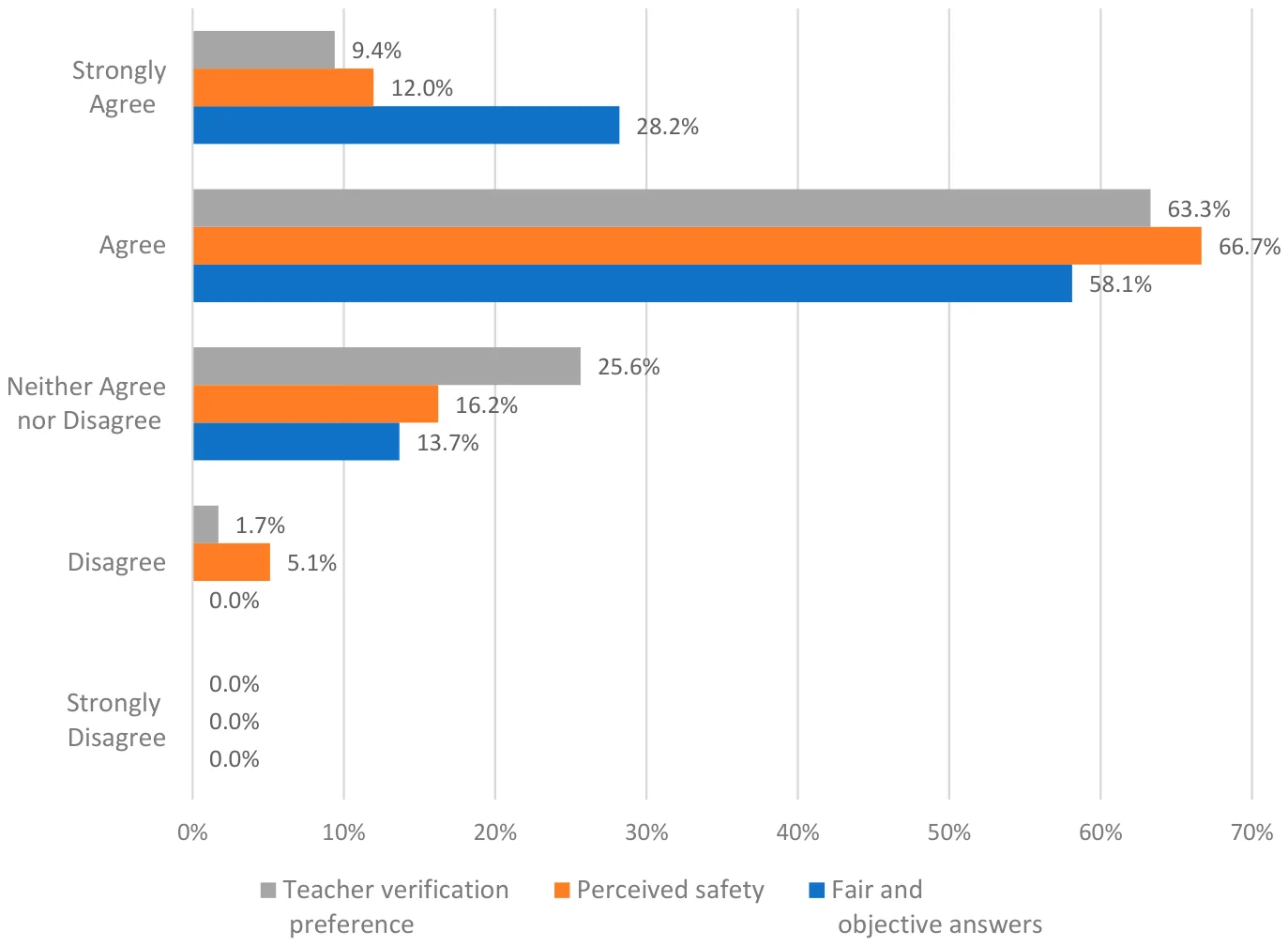
Learners’ perceptions of trust, safety, and educator verification when using the e-Teacher Assistant.

As shown in Figure 12, 86.3% of participants agreed or strongly agreed that the e-Teacher Assistant provided fair and objective answers, with 58.1% selecting Agree and 28.2% selecting Strongly Agree. The remaining 13.7% provided a neutral response, while no disagreement was recorded.

Regarding perceived safety, 78.6% of participants selected Agree or Strongly Agree, comprising 66.7% and 12.0%, respectively. A further 16.2% provided a neutral response, while 5.1% selected Disagree. No participants selected Strongly Disagree.

For preference for educator verification, 72.6% of participants selected Agree or Strongly Agree, with 63.2% selecting Agree and 9.4% selecting Strongly Agree. Neutral responses accounted for 25.6%, while 1.7% selected Disagree. No Strongly Disagree responses were recorded.

### 5.6. Open-Ended Responses

Learners’ responses to the two open-ended questionnaire questions were analysed using reflexive thematic analysis, as described in Section 4.3.

Analysis of responses to the question concerning the aspects learners valued most resulted in five main themes: (1) AI-assisted immediate learning support, (2) learning from mistakes, practice, and reassessment, (3) personalisation and targeted adaptive support, (4) usability, accessibility, and flexibility, and (5) progress awareness and integrated learning.

A prominent theme was the availability of immediate support through the AI Assistant. Learners frequently referred to the assistant itself, the ability to ask questions and obtain explanations at any time, and the availability of contextual assistance during learning activities and examinations. Responses also highlighted the usefulness of receiving examples and clarifications while working with the learning material.

A second recurring theme related to learning through mistakes, repeated practice, and reassessment. Learners responded positively to the opportunity to review incorrect responses, understand why errors had occurred, practise further, and repeat examinations in order to improve their performance. Several responses explicitly described mistakes as opportunities for further learning rather than as final outcomes.

Personalisation and targeted adaptive support formed a third theme. Learners referred to the system’s ability to identify areas of difficulty, adapt support to their individual needs, provide targeted questions, and offer what some participants described as a “tailor-made” learning experience.

A fourth theme related to usability, accessibility, and flexibility. Participants described the application as easy to use and understandable and valued immediate access to learning material and the possibility of engaging with the system at times that suited their individual study needs. Some responses also referred to support for organising learning material and study activities.

The fifth theme concerned progress awareness and the integration of learning activities. Learners valued being able to monitor their performance across chapters, review previous mistakes, and receive recommendations concerning further theory study or practice. Some participants also referred positively to the integration of theoretical study, practice, assessment, and AI-assisted support within the same learning environment.

Responses to the second open-ended question showed that many learners did not identify a specific aspect requiring improvement, instead providing responses such as “nothing,” “everything was fine,” or indicating that they had not identified a problem. Among participants who proposed improvements, suggestions primarily concerned the user interface and greater visual differentiation between pages, additional accessibility options such as language selection and remote access, and improved system response speed. A small number of additional comments concerned specific functionality and the need for further testing under authentic conditions. One participant explicitly raised a concern regarding the protection of personal data.

### 5.7. Teachers’ Evaluation of the Teacher Model

To complement the learners’ evaluation presented in the previous sections, the perspectives of the four educators who participated in the evaluation of the e-Teacher Assistant were also examined. Their evaluation focused on the perceived usefulness of the system for monitoring learner progress, identifying learning needs, understanding the instructional recommendations provided by the Teacher Model, and supporting instructional decision-making. Given the pronounced sample asymmetry between the learner cohort (N=117) and the participating educators (n=4), the educator-facing data are strictly treated as exploratory, preliminary qualitative indicators rather than generalizable quantitative conclusions. The Likert-scale responses serve as structured thematic probes that contextualize the educators’ open-ended feedback within this specific vocational deployment.

**Figure 13:**
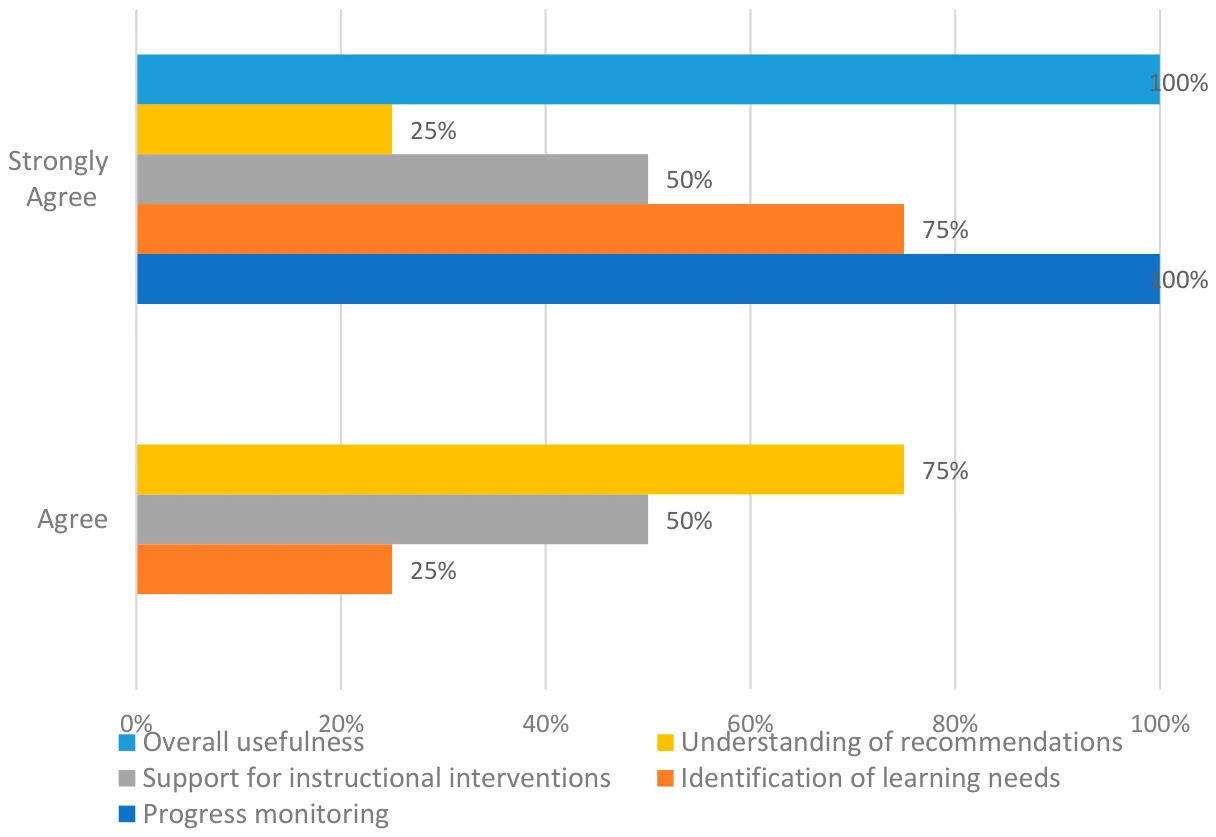
Teachers’ perceptions of the Teacher Model and its support for learning analytics and instructional decision-making.

As shown in Figure 13, all four educators evaluated the system positively across the examined aspects, with no neutral or negative responses recorded. All four educators (4/4) strongly agreed that the e-Teacher Assistant was useful overall and supported the monitoring of learner progress.

Regarding the identification of learning needs, three educators (3/4) strongly agreed that the system helped identify learners’ difficulties and needs, while one (1/4) agreed. Support for instructional interventions was also evaluated positively by all educators, with two educators (2/4) selecting “Strongly Agree” and two (2/4) selecting “Agree”. Regarding understanding of the instructional recommendations provided by the Teacher Model, one educator (1/4) strongly agreed and three (3/4) agreed.

The open-ended responses provided further insight into these evaluations. Educators particularly valued the ability to monitor both individual learner progress and overall class progress, identify specific learning difficulties, and provide targeted practice and support. They also highlighted the practical value of the Teacher Model in reducing time spent on routine monitoring and assessment-related tasks, allowing greater attention to lesson preparation and interaction with learners. The responses further suggested that the system’s exercises, feedback, and learning materials supported classroom interaction and instructional practice.

Suggestions for improvement focused primarily on further personalisation and system responsiveness. Two educators suggested that the system could better identify the underlying reasons for learners’ errors and adapt explanations and activities accordingly. They also suggested using the outcomes of previous interventions to improve subsequent recommendations progressively. Faster AI response times, particularly during answer analysis and the generation of personalised exercises, were also identified as a potential area for improvement.

### 5.8. Relationships Between Dimensions and Group Comparisons

The internal consistency reliability of the questionnaire was assessed using Cronbach’s alpha coefficient for each multi-item evaluation dimension. For the two-item Motivation and Persistence dimension, the Spearman–Brown coefficient was also calculated (rSB = 0.876; inter-item Pearson r = 0.779), confirming the reliability estimate obtained with Cronbach’s alpha. The results suggested high to excellent internal consistency for six of the seven evaluated dimensions, with Cronbach’s α coefficients ranging from 0.873 to 0.970. The Trust, Safety, and Human Oversight dimension showed comparatively lower internal consistency (α=0.668). An item-level inspection confirmed that this psychometric attenuation stems from structural dimensionality: items T1 (‘fair and objective answers’) and T2 (‘feel safe while using it’) exhibited a moderate positive inter-item correlation (r=0.542,p<0.001), yielding an acceptable two-item reliability (α=0.702, Spearman–Brown $r_{SB}=0.703$). In contrast, item T3 (‘preference to verify answers with the teacher’) correlated weakly with T1 (r=0.281,p=0.002) and T2 (r=0.314,p<0.001), reflecting a distinct construct related to human-in-the-loop oversight rather than perceived system benevolence or algorithmic safety. Removing T3 increases the scale’s internal consistency to α=0.702. The reliability coefficients for all evaluation dimensions are presented in Table 3.

To complement the descriptive findings for the seven questionnaire dimensions, relationships among the principal evaluation dimensions were examined using Spearman’s rank-order correlation coefficients. The resulting correlation matrix is presented in Table 4.

As shown in Table 3, all examined dimensions were positively and statistically significantly correlated (p<0.001). The strongest association was observed between Motivation and Persistence and Emotional Support and Interaction (ρ=0.877,p<0.001). To examine potential multicollinearity between these two highly correlated dimensions, collinearity diagnostics were calculated. The resulting Variance Inflation Factor (VIF = 4.35, tolerance = 0.230) remained below the conservative threshold of 5.0 (and well below the common threshold of 10.0), indicating that while they share substantial variance, they remain statistically distinguishable within regression and comparative frameworks. Conceptually, Motivation and Persistence captures goal-directed cognitive drive and perceived comfort in making errors, whereas Emotional Support and Interaction reflects the affective tone, perceived benevolence, and immediate relational feedback provided by the conversational AI. Strong positive correlations were also observed between Usability and Ease of Use and Learning Support (ρ=0.831,p<0.001), between Perceived Usefulness, Personalisation and Acceptance and Motivation and Persistence (ρ=0.819,p<0.001), and between Perceived Usefulness, Personalisation and Acceptance and Learning Support (ρ=0.803,p<0.001).

To investigate whether learners’ prior familiarity with digital learning tools was associated with their evaluations of the proposed system, participants were grouped into frequent or very frequent users (n=76), occasional users (n=32), and rare or non-users (n=9). Kruskal–Wallis H tests revealed statistically significant differences across the three experience groups for all seven evaluation dimensions: Usability and Ease of Use ($H\left(2\right)=25.475,p<0.001,\eta_{H}^{2}=0.206,95\%\;\mathrm{CI}\;\left[0.089,0.341\right]$), Learning Support ($H\left(2\right)=32.787,p<0.001,\eta_{H}^{2}=0.270,95\%\;\mathrm{CI}\;\left[0.142,0.409\right]$), Motivation and Persistence ($H\left(2\right)=20.251,p<0.001,\eta_{H}^{2}=0.160,95\%\;\mathrm{CI}\;\left[0.052,0.292\right]$), Emotional Support and Interaction ($H\left(2\right)=19.162,p<0.001,\eta_{H}^{2}=0.151,95\%\;\mathrm{CI}\;\left[0.046,0.281\right]$), Perceived Usefulness, Personalisation and Acceptance $H\left(2\right)=28.389,p<0.001,\eta_{H}^{2}=0.231,95\%\;\mathrm{CI}\;\left[0.109,0.368\right]$, Teacher Support and Learning Analytics ($H\left(2\right)=11.804,p=0.003,\eta_{H}^{2}=0.086,95\%\;\mathrm{CI}\;\left[0.012,0.198\right]$), and Trust, Safety, and Human Oversight ($H\left(2\right)=32.958,p<0.001,\eta_{H}^{2}=0.272,95\%\;\mathrm{CI}\;\left[0.144,0.411\right]$).

Pairwise post hoc comparisons were conducted using exact Mann–Whitney U tests with a Bonferroni-adjusted threshold of p<0.0167. Table 5 provides the complete breakdown of group medians, interquartile ranges (IQR), exact U statistics, adjusted p-values, and rank-biserial effect sizes (r).

To verify that these familiarity-based differences were not confounded by instructor-specific delivery styles or classroom clustering, participant distribution across the four teaching cohorts was cross-tabulated against digital experience levels. The four educator cohorts comprised 31, 29, 29, and 28 learners, respectively. A chi-square test of independence confirmed that the distribution of digital familiarity (frequent, occasional, rare/non-user) did not differ significantly across the four teacher classrooms ($\chi^{2}\left(6\right)=2.84,p=0.828$). Frequent users accounted for 61.3–67.9% of each class cohort, occasional users represented 24.1–31.0%, and rare/non-users represented 6.9–10.3%. This balanced distribution indicates that the observed perceptual differences across experience tiers reflect genuine learner-level digital readiness rather than instructor-specific pedagogical or grading influences.

In contrast, comparisons between occasional users and rare or non-users revealed no statistically significant differences in Usability and Ease of Use, Learning Support, Motivation and Persistence, Emotional Support and Interaction, Perceived Usefulness, Personalisation and Acceptance, or Teacher Support and Learning Analytics. A statistically significant difference between these two groups was observed only for Trust, Safety, and Human Oversight (U=45.000,p=0.001), with occasional users showing higher mean ranks than rare or non-users.

### 5.9. Log Data Analytics from the Learning Record Store

#### 5.9.1. Learning Engagement

The behavioral analysis evaluated interaction traces generated by the cohort of 117 learners across the six curriculum chapters. Data hygiene filtering was applied to ensure that analyzed interactions reflected authentic, completed instructional engagement rather than transient navigations: from an initial raw pool of 188 logged learner–chapter sessions, 25 records (13.3%) were excluded due to: (a) zero or negligible dwell time (<2 min) indicating accidental clicks (n=14), (b) premature session termination prior to attempting the mandatory embedded mini-quiz (n=8), and (c) network socket disconnects yielding corrupted LRS payloads (n=3). The remaining 163 qualified learner–chapter records formed the analytic dataset.

The distribution of chapter participation per learner was as follows: 79 learners (67.5%) contributed 1 qualified chapter, 30 learners (25.6%) completed 2 chapters, and 8 learners (6.8%) completed 3 chapters. Across the course curriculum, the 163 records were distributed across chapters as follows: Chapter 1 (n=28), Chapter 2 (n=30), Chapter 3 (n=28), Chapter 4 (n=22), Chapter 5 (n=34), and Chapter 6 (n=21). All learner interactions with the theoretical learning environment were automatically recorded and used to derive objective learning-engagement indicators. Because the six course chapters contained different numbers of learning topics, a normalised engagement indicator—study time per topic—was also computed to enable comparisons across chapters.

Table 6 summarises objective learning-engagement indicators derived from the Learning Record Store (LRS). Theory study time was calculated as the cumulative duration of completed theory-view events. Across the analysed learner–chapter records, learners spent an average of 48.21 +/− 36.14 min studying the theoretical content. Course chapters ranged from 2 to 14 learning topics.

Learners completed the theoretical material in an average of 1.91 +/− 2.47 theory study sessions (median=1.00, range=1−25). Most learner–chapter records (68.1%) were completed during a single study session, while 81.0% required no more than two sessions. The number of theory study sessions ranged from 1 to 25 across learner–chapter records.

Learners asked an average of 3.17 +/− 3.04 AI questions per learner–chapter record (median=3.00, range=0−27), distributed across 1.74 +/− 2.45 AI sessions (median=1.00, range=0−25).

The content-regeneration functionality was used a mean of 0.56 +/− 0.85 requests per learner–chapter record (median=0.00, range=0−4). A median of zero suggests that at least half of the learner–chapter records contained no content-regeneration request, while others included between one and four requests.

Learners spent an average of 10.90 +/− 5.24 min per completed topic (median=9.30 min, range=4.87−35.01). Study time per topic was reported in addition to total chapter study time because the six instructional chapters contained different numbers of learning topics.

Overall, the LRS-based engagement indicators showed variability in study duration, number of study sessions, AI-assisted interactions, and content-regeneration requests. Because progression to the practical activity or final examination required completion of the theoretical content and the embedded mini-quiz, the analysed learner–chapter records corresponded to completed theoretical learning sessions rather than partial attempts.

#### 5.9.2. Examination Analytics

Table 7 summarises the descriptive statistics for the examination analytics indicators. Examination grades were originally scored on a 0–20 scale and are reported here in their raw form; the fuzzy inference mechanism normalises these values to the [0, 1] interval as input to the Examination Performance variable (Section Formal Specification of the Fuzzy Inference Mechanism). Learners completed an average of 1.50 ± 0.97 examination attempts (median=1.00, range=1−4). The first examination grade averaged 15.26 ± 0.96, whereas the final and best examination grades averaged 16.14 ± 1.57 and 16.15 ± 1.58, respectively. The first grade represents learners’ initial performance, the final grade corresponds to the last recorded examination attempt, and the best grade represents the highest score achieved across all attempts.

The distribution of examination attempts showed that 73.62% of learner–chapter records involved a single examination attempt, whereas 26.38% involved repeated attempts. Among the 43 learner–chapter records with more than one examination attempt, 40 (93.02%) achieved a higher final examination grade than on their first attempt. The remaining three records (6.98%) did not show improvement, including two with unchanged performance and one with a lower final grade.

Examination repetition was mandatory for learners who did not achieve a passing grade, whereas learners who had already passed could also choose to repeat the examination voluntarily to improve their score. Repeated examination attempts therefore included both mandatory and voluntary reassessment. Crucially, these performance gains cannot be attributed to a simple re-testing or test-familiarity effect. Under the system’s pedagogical workflow, reassessment was not an isolated, repetitive event; rather, access to a subsequent examination attempt was strictly conditioned upon engaging with tailored remediation pathways. Depending on initial diagnostic flags (such as Needs Retheory or Needs More Practice), learners were systematically directed to review targeted theoretical concepts and complete individualized formative practice modules prior to reassessment, ensuring that score progression reflected cognitive remediation rather than procedural recall.

Within the adaptive learning workflow, learners completed the theoretical learning phase and the embedded mini-quiz before becoming eligible for practical activities or the chapter examination. Following each examination, the system generated adaptive recommendations based on learners’ performance. Learners classified as Very Weak were required to revisit the theoretical learning material and complete additional practical learning activities before reattempting the examination, whereas learners classified as Weak were required to complete additional practical exercises before reassessment.

#### 5.9.3. AI Assistant Usage and Examination Support

In addition to the examination analytics, learner interactions with the AI Assistant were examined. Two distinct forms of AI-supported interaction were available within the proposed framework: a general AI Assistant during theoretical study and contextual AI assistance embedded within examinations. The former supported learner-initiated questions and alternative content generation during theory study, whereas the latter provided contextual guidance for eligible examination questions.

During the theoretical learning phase, AI-related engagement indicators—including AI questions, AI sessions, and content regenerations—were presented as part of the overall learning-engagement analysis (Section 5.9.1, Table 6). These indicators describe learners’ voluntary interactions with the AI Assistant while studying the theoretical material.

The present section focuses on AI-supported interactions that occurred during examinations. The examination dataset comprised 163 learner–chapter records, of which 105 (64.42%) involved at least one AI-assisted examination interaction, whereas the remaining 58 (35.58%) contained no recorded AI-supported examination interaction. Across all instructional chapters, learners requested assistance for 197 examination questions, distributed across 190 help sessions and generating 246 dialogue turns in total.

The use of contextual examination assistance varied across instructional chapters. AI-supported help was recorded in 32.14% of Chapter 1 examination records, 60.00% in Chapter 2, 50.00% in Chapter 3, 90.91% in Chapter 4, 73.53% in Chapter 5, and 83.33% in Chapter 6.

Among the 105 learner–chapter records in which examination assistance was used (Table 8), learners had an average of 9.40 ± 2.31 examination questions eligible for AI support (median=11, range=5−11). Assistance was requested for an average of 1.88 ± 1.18 questions (median = 1, range = 1–6), corresponding to a mean help-usage ratio of 0.215 ± 0.149 within this help-active subgroup (n=105). Specifically, this indicates that among the 105 records where AI assistance was utilized during examinations, learners sought help on approximately 21.5% of the eligible assessment questions on average (whereas across the complete sample of N=163 records, the unconditional help-usage ratio across all attempts averages 0.138 ± 0.156).

Within these 105 records, learners completed an average of 1.81 ± 1.14 help sessions (median=1, range=1−6) and 2.34 ± 1.79 dialogue turns (median=2, range=1−9). Only two learner–chapter records contained off-topic interactions, each consisting of a single off-topic dialogue turn.

#### 5.9.4. Triangulation of Learning Analytics and Learner Perceptions

The evaluation of the proposed e-Teacher Assistant combined two complementary sources of evidence. Learners’ perceptions were collected through the post-intervention questionnaire, whereas interaction data were recorded through the Learning Record Store (LRS). These two sources were examined together to compare learners’ reported perceptions with their recorded interaction behaviour during the intervention.

The questionnaire results showed predominantly positive perceptions regarding usability, learning support, personalisation, teacher support, and acceptance of the proposed system. The LRS data provided complementary information on learners’ actual engagement with the learning environment, examination behaviour, and use of AI-assisted support.

The engagement analytics presented in Section 5.9.1 showed that learners interacted with the theoretical learning environment through study sessions, AI questions, AI sessions, and content-regeneration requests. These behavioural indicators were considered alongside questionnaire responses related to learning support, independent learning, and interaction with the system.

The examination analytics presented in Section 5.9.2 showed that 26.38% of learner–chapter records involved repeated examination attempts, with 93.02% of repeated records exhibiting score gains between initial and final attempts. Rather than demonstrating an unmediated testing effect, this behavioural pattern converges with learners’ self-reported perceptions regarding reflection on errors and iterative practice. The recorded score gains reflect learners’ progression through the closed-loop remediation workflow, wherein diagnostic difficulty flags required students to consolidate foundational knowledge through adaptive practice and theory revisits before unlocking re-examination opportunities.

The AI usage analytics presented in Section 5.9.3 provided additional behavioural data on interactions with the AI Assistant. During examinations, contextual AI assistance was actively engaged in 64.42% of learner–chapter records (105 of 163), with a mean help-usage ratio of 0.215 restricted specifically to those 105 help-active assessment records. These indicators were considered alongside questionnaire responses concerning the usefulness of the AI Assistant, independent learning, and interaction with the system.

Learners’ perceptions of teacher support were also considered together with LRS-based indicators generated by the Teacher Model. Questionnaire responses addressed the identification of individual and class-level learning difficulties and lesson adaptation, while the recorded analytics included information on learner engagement, examination behaviour, AI-assisted interactions, and chapter-level progress.

## 6. Discussion

The present study evaluated the e-Teacher Assistant in an authentic vocational education context, with particular emphasis on learners’ perceptions of usability, learning support, personalisation, autonomous learning, trust in AI, and the role of the Teacher Model in supporting pedagogical monitoring and decision-making. In addition to questionnaire-based evaluation, Learning Record Store (LRS) data provided behavioural evidence regarding learner engagement, examination activity, and use of AI-assisted support. Taken together, the findings provide a multidimensional view of how learners and educators experienced the proposed human-centred and explainable educational framework.

### 6.1. Usability, Usefulness, and Perceived Educational Value (RQ1)

With respect to RQ1, the findings suggest that learners generally perceived the e-Teacher Assistant as usable, useful, and supportive of the learning process. Positive evaluations were particularly evident for ease of use, clarity of instructions, feedback usefulness, interaction with the AI Assistant, and recommendation for wider use. These findings are relevant because usability and perceived usefulness appear to be important conditions for the practical adoption of AI-supported educational systems. A technically advanced learning environment is unlikely to provide meaningful educational value if learners experience difficulties in navigating the system or understanding how to use its support mechanisms.

The strong association observed between Usability and Ease of Use and Learning Support (ρ=0.831,p<0.001) further suggests that learners’ perceptions of the system’s educational value were closely connected to their experience of interacting with it. Similarly, Perceived Usefulness, Personalisation and Acceptance was strongly associated with both Learning Support (ρ=0.803,p<0.001) and Motivation and Persistence (ρ=0.819,p<0.001). These relationships suggest that usability, perceived usefulness, and educational support were not experienced as independent characteristics of the system, but rather as interconnected dimensions of the overall learning experience.

This pattern is consistent with the broader literature reviewed in this study, which emphasises that the educational value of AI-based systems depends not only on their technical capabilities but also on factors such as the usefulness and clarity of feedback, learner motivation, trust, and alignment with pedagogical principles [25,26,27]. The findings therefore support the view that educational AI is more likely to be experienced positively when technical functionality is combined with an accessible and pedagogically meaningful learner experience.

The qualitative findings provide additional support for this interpretation. Learners frequently highlighted the dual-mode interaction of the AI Assistant—specifically praising the accessible analogies during theoretical preparation and the supportive Socratic prompting during examinations without feeling judged or penalized. The strict bounding of LLM responses via RAG and curriculum-grounded guardrails proved instrumental in preventing both hallucinated feedback and answer leaking, validating the integration of generative AI into formal assessment pipelines. At the same time, suggestions for improvement focused mainly on interface design, additional language options, system responsiveness, and data-protection concerns. These reservations were primarily related to implementation and user-experience considerations rather than to the overall perceived value of the framework.

### 6.2. Autonomous Learning, Reflection on Mistakes, and Persistence (RQ2)

RQ2 examined whether learners perceived the system as supporting autonomous learning, active engagement, and reflection on mistakes. The questionnaire findings showed positive perceptions across these areas. Learners reported that the system helped them work independently, learn from mistakes, and continue their learning efforts after incorrect responses. Particularly strong positive evaluations were observed for learning from mistakes and the usefulness of interaction with the AI Assistant.

A notable finding was the strong correlation between Motivation and Persistence and Emotional Support and Interaction (ρ=0.877,p<0.001), which was the strongest association observed among all questionnaire dimensions. This suggests that learners who experienced interaction with the system as supportive also tended to report greater motivation and persistence. Such a strong association indicates that learners experienced motivation, persistence, and emotional reassurance as closely intertwined facets of a cohesive, error-supportive learning environment. At the same time, this high shared variance highlights the issue of discriminant validity between cognitive persistence (e.g., willingness to rework flawed problems) and affective reassurance (e.g., absence of punitive perception during failure). In our framework, these dimensions were designed to probe complementary sides of formative assessment: the functional intention to persist versus the affective climate created by the conversational agent. Nevertheless, the empirical proximity of these dimensions suggests that learners may perceive them holistically as an integrated affective–motivational experience rather than as separate cognitive and affective constructs.

The LRS data offer complementary behavioural evidence. Learners interacted with the AI Assistant during theoretical study, asking an average of 3.17 questions per learner–chapter record, while examination-related assistance was used in 64.42% of learner–chapter records. At the same time, the mean help-usage ratio during examinations was 0.215 specifically among the 105 records where help was invoked, demonstrating that even when students opted to use AI support during testing, they restricted assistance to roughly 21.5% of eligible items on average rather than relying on automated guidance continuously. This pattern suggests that learners engaged in productive, selective help-seeking rather than continuous technological crutching, aligning with the framework’s pedagogical intent. Because Autonomy normalizes for varying chapter-level question pools and Uncertainty isolates conversational depth from invocation frequency, the system mathematically accommodates targeted assistance without conflating it with cognitive deficiency or low autonomy. The sensitivity analysis in the Formal Specification of the Fuzzy Inference Mechanism Section corroborates this design: targeted, low-turn interventions preserve the student’s proficiency level, ensuring that learners are not penalized for using AI as a constructive scaffold.

The examination analytics provide a further complementary perspective. Among the 43 learner–chapter records involving repeated examination attempts, 40 (93.02%) achieved a higher final examination grade than on the initial attempt. However, these gains must not be conflated with an unmediated testing effect, in which repeated exposure to assessment items artificially inflates post-test scores. Reassessment within the e-Teacher Assistant was explicitly coupled with an adaptive remediation protocol: learners failing to demonstrate mastery were locked out of immediate re-testing and mandated to engage in structured remediation—namely, revisiting weak theoretical subunits and completing adaptive, error-targeted practice modules. Consequently, performance improvement is properly attributed to this comprehensive, scaffolded remediation workflow rather than re-examination in isolation.

These findings are consistent with the design of the Student Model, which combines performance information with engagement, interaction, practice, and adaptive feedback mechanisms. In this respect, the findings provide initial support for the view that adaptive learning support can be integrated with reflection on errors and repeated practice in ways that learners perceive as supportive rather than punitive.

### 6.3. Teacher Model, Learning Analytics, and Human-Centred Decision Support (RQ3)

RQ3 focused on how learners and educators perceived the role of the Teacher Model in supporting pedagogical monitoring and decision-making. The learner evaluation showed consistently positive perceptions regarding the system’s ability to support the identification of individual learning difficulties, class-level difficulties, and lesson adaptation. These perceptions were also reflected in the educator evaluation.

All four participating educators evaluated the Teacher Model positively across the examined dimensions. All educators strongly agreed that the system was useful overall and supported progress monitoring. They also positively evaluated its contribution to identifying learning needs, understanding the instructional recommendations provided by the Teacher Model, and supporting instructional interventions. Reflecting the sample size (n=4), these teacher findings must not be interpreted as statistically generalizable evidence of broad pedagogical efficacy. Instead, they provide preliminary, context-bound qualitative indications of how domain practitioners interact with and interpret actionable learning analytics during everyday classroom orchestration.

The educators’ qualitative responses further indicated that they valued the ability to monitor both individual learners and the overall class, identify learning difficulties, and provide more targeted support. They also reported that the Teacher Model could reduce time spent on routine monitoring and assessment-related activities, potentially allowing greater attention to lesson preparation and direct interaction with learners. At the same time, educators identified areas for further improvement, including deeper identification of the causes underlying learner errors, greater adaptation of explanations and activities, and faster AI response times.

These findings speak directly to the research gap identified in the study. Much of the existing literature on AI-supported education focuses primarily on learner-facing support, performance prediction, or static identification of at-risk learners, with comparatively less attention paid to interpretable, continuous, teacher-facing pedagogical decision support [12,27,29,33]. The proposed Teacher Model contributes to this area by providing linguistically transparent summaries derived from dynamic fuzzy rules, offering educators an interpretable window into the reasoning behind automated recommendation flags. However, because the present study did not administer a dedicated, formal XAI evaluation instrument (such as the Explanation Satisfaction Scale), these interpretability benefits must be interpreted with caution. While teachers reported high descriptive satisfaction and comprehension of the recommendations during everyday orchestration, formal cognitive validation of the explanations remains an essential avenue for empirical verification. Similarly, while the participating educators reported positive perceptions regarding instructional intervention support, these observational findings reflect perceived utility and user acceptance rather than verified improvements in objective teacher decision quality or decision accuracy. Demonstrating whether such decision-support dashboards lead to objectively superior instructional choices would require comparative trial designs evaluating pedagogical outcomes against unassisted teaching baselines.

The convergence between learners’ perceptions and the LRS-based analytics is particularly relevant in this context. Learners perceived the system as supporting the teacher’s ability to understand learning difficulties and adapt instruction, while the Teacher Model generated objective information concerning engagement, examination behaviour, AI-assisted interactions, and chapter-level progress. This combination provides a broader empirical basis than either self-report or usage analytics alone and supports the use of LRS-based learning analytics as a mechanism for informing pedagogical decision-making.

Importantly, the findings should not be interpreted as supporting automated pedagogical decision-making. The architecture of the proposed system was explicitly designed around a human-in-the-loop approach in which the educator retains responsibility for instructional decisions, progression, assessment, and intervention. The Teacher Model therefore functions as a decision-support mechanism rather than as an autonomous decision-maker. This distinction is especially important in light of broader concerns regarding transparency, interpretability, and human oversight in AI-supported educational environments.

### 6.4. Trust in AI and the Continuing Role of the Educator (RQ1/RQ3)

The trust-related findings provide further support for the human-centred orientation of the proposed framework. Most learners viewed the fairness and objectivity of the system positively and reported feeling safe when using it. At the same time, a substantial proportion also preferred to verify the system’s responses with the teacher.

Rather than representing a contradiction, this pattern suggests that trust in AI-supported educational systems may coexist with a continuing preference for human verification and pedagogical contextualisation. Learners may therefore accept AI-generated support while still recognising the educator as an important source of judgement, explanation, and confirmation. This is particularly relevant in educational contexts, where the appropriateness of feedback and recommendations cannot always be determined exclusively through computational mechanisms.

The comparatively lower internal consistency of the initial Trust, Safety, and Human Oversight dimension (α=0.668) warrants structural clarification. Psychometrically, our item-level analysis demonstrates that perceived algorithmic safety (T1, T2) and preference for teacher verification (T3) represent two distinct, complementary dimensions rather than a single homogeneous latent trait. System trust reflects confidence in the AI’s immediate outputs, whereas teacher verification reflects epistemic vigilance and reliance on pedagogical authority. In human-centered learning environments, a high desire for teacher verification does not necessarily indicate distrust in technology; rather, it underscores the learners’ recognition of the educator as the final arbiter of domain expertise. Consequently, treating these dimensions as structurally separate constructs provides both greater psychometric clarity and a more nuanced understanding of human–AI educational partnerships.

This result is consistent with the conceptual orientation of the proposed system. The purpose of the e-Teacher Assistant is not to replace the educator but to complement pedagogical judgement through transparent learner analytics and interpretable recommendations. The continued preference for teacher verification therefore aligns with, rather than undermines, the intended human-in-the-loop architecture.

### 6.5. Association with Prior Digital Learning Experience (RQ4)

RQ4 examined whether prior familiarity with digital learning tools was associated with learners’ evaluations of the adaptive and explainable features of the e-Teacher Assistant. The findings revealed statistically significant differences across prior-experience groups for all seven evaluation dimensions. Frequent or very frequent users reported significantly more positive evaluations than both occasional users and rare or non-users across all dimensions.

These results suggest that familiarity with digital learning environments may be related to how readily learners engage with and evaluate AI-supported educational technologies. Learners with more extensive previous experience may be more comfortable navigating digital environments, interpreting automated feedback, and interacting with adaptive or AI-based support mechanisms. In contrast, learners with limited previous exposure may require additional familiarisation, orientation, or scaffolding before they can engage with such systems in the same way.

Trust, Safety, and Human Oversight showed the clearest differentiation across prior-experience groups. Unlike the other six dimensions, a statistically significant difference was also observed between occasional users and rare or non-users. This finding suggests that prior digital experience may be particularly relevant to learners’ sense of confidence and trust when interacting with AI-mediated educational support.

However, these associations should not be interpreted as indicating that learners with lower digital experience are inherently less suited to AI-supported learning. Rather, they highlight the importance of digital readiness when introducing intelligent educational technologies. In vocational education settings, where learners may enter courses with heterogeneous levels of prior technological experience, implementation strategies may need to include structured orientation and opportunities to develop familiarity with AI-supported learning environments. Given the small number of rare or non-users (n = 9), findings involving this subgroup should be interpreted cautiously.

### 6.6. Convergence of Perception and Behavioural Analytics

A key strength of the evaluation is the use of both subjective questionnaire data and objectively recorded LRS interaction data. These two sources of evidence capture different aspects of the educational experience: questionnaire responses describe how learners perceived the system, whereas LRS data provide information about how learners interacted with the learning environment.

Several areas of convergence were observed. Positive perceptions of learning support and autonomous learning were accompanied by recorded engagement with theoretical content and AI-assisted support. Learners’ perceptions regarding reflection on mistakes and persistence were complemented by repeated learning and examination activity within the adaptive remediation workflow. Similarly, questionnaire-based perceptions of teacher support were accompanied by the generation of learner- and class-level analytics through the Teacher Model.

This convergence strengthens the interpretation of the evaluation because the findings are not based solely on self-reported perceptions. At the same time, the LRS data should not be treated as direct evidence of learning effectiveness. Usage frequency, repeated attempts, and AI interaction traces describe observational engagement patterns within the remediation workflow, but they do not provide evidence of causal learning gains. In the absence of a counterfactual control group receiving alternative or unassisted instruction, performance improvements across repeated assessments cannot be causally attributed to the system over general instructional progression or learning over time. Nevertheless, combining perception data with behavioural analytics provides a more comprehensive empirical picture of the system’s use under authentic classroom conditions.

Overall, the findings provide initial support for the central design proposition of the study: that LRS-based learning analytics, interpretable learner modelling, adaptive AI-assisted support, and teacher-facing decision support can be integrated within a unified educational framework. The results also reinforce the importance of maintaining human pedagogical oversight. Rather than positioning AI as an autonomous instructional agent, the findings support a hybrid model in which AI provides personalised support and interpretable analytics while the educator retains responsibility for pedagogical judgement and decision-making.

## 7. Conclusions and Future Work

### 7.1. Limitations

Several limitations should be considered when interpreting the findings. First, the evaluation was conducted at a single vocational-education institution with a single cohort of 117 learners enrolled in a Computer Networks course. This limits the generalisability of the findings to other subject areas, educational levels, and institutional settings. Replication across multiple institutions and educational contexts is therefore needed to strengthen external validity. Furthermore, a substantial sample asymmetry exists between the student cohort (N=117) and the teaching staff (n=4). While the educator sample encompasses all instructors responsible for the delivered curriculum, evaluating decision-support tools with four teachers necessarily limits the findings to exploratory qualitative insights. Future research must involve multi-institutional educator cohorts to enable inferential, psychometrically validated assessments of teacher dashboard adoption.

Second, the evaluation design did not include a control group receiving conventional instruction or pre-test/post-test measures of learning gains. Consequently, the study does not permit causal claims regarding the effect of the e-Teacher Assistant on learning outcomes. The findings primarily concern learners’ perceptions, acceptance, usage patterns, and the feasibility of deploying the system in an authentic vocational-education setting. Similarly, while score gains across repeated attempts provide operational evidence of the system’s remediation loop, the absence of a control group receiving repeated tests without adaptive intervention means the exact effect size of the remediation workflow versus test-taking familiarity cannot be completely isolated, representing a key direction for controlled experimental follow-up.

Third, the intervention was conducted with active involvement from the educators, who introduced curriculum units, guided learners’ reflection on mistakes, and provided pedagogical scaffolding throughout the learning process. As a result, the contribution of the e-Teacher Assistant cannot be fully separated from that of the educators’ instructional practice. Although this is consistent with the intended human-in-the-loop design of the system, it limits the extent to which observed outcomes can be attributed to the technological component alone.

Fourth, much of the evaluation evidence was based on self-reported questionnaire data, which may have been influenced by social-desirability and novelty effects. The LRS-based analytics presented in Section 5.9 provided complementary objective evidence regarding learner interaction and usage behaviour, but they do not replace controlled experimental or quasi-experimental evaluation.

Fifth, while the fuzzy inference engine and rule-to-text transformation were designed to foster algorithmic transparency, the system’s explainability component was not evaluated using standardized, formal XAI psychometric batteries. The evidence reported herein is derived from post-intervention teacher Likert items and open-ended feedback regarding dashboard utility, rather than controlled psychometric assessments of explanation goodness, mental-model fidelity, or counterfactual understanding. Consequently, our findings should be understood as preliminary empirical indications of practical interpretability rather than validated proof of cognitive explanation efficacy. Subsequent studies will formally integrate standardized frameworks, such as the Explanation Satisfaction Scale and user trust batteries, to rigorously quantify explanation quality.

Finally, although the questionnaire dimensions were informed by established instruments, including TAM, SUS, UEQ, and recent XAI-in-education evaluation approaches, the questionnaire was tailored to the specific context of the present study. This may limit cross-study comparability, and future evaluations should consider combining context-specific measures with standardised instruments. Additionally, the internal consistency of the composite Trust, Safety, and Human Oversight construct fell marginally below the standard threshold (α=0.668). As revealed by item-level diagnostics, this attenuation was driven by bundling perceived algorithmic safety and objectivity with epistemic preference for teacher verification. These represent structurally distinct constructs that operate under different psychological mechanisms. Future iterations will formally decouple these items into two independent scales—namely, ‘System Trust & Safety’ and ‘Epistemic Human Reliance’—and subject them to formal Confirmatory Factor Analysis (CFA) across diverse cohorts. In addition, the strong correlations observed among several questionnaire dimensions may indicate a degree of conceptual overlap between the evaluated domains. Accordingly, these dimensions should be interpreted as theoretically informed aspects of learner experience rather than as fully validated independent latent constructs. In particular, the strong inter-correlation between Motivation and Persistence and Emotional Support and Interaction (ρ=0.877, p<0.001) raises legitimate concerns regarding empirical discriminant validity and multicollinearity. Although collinearity diagnostics indicated acceptable separation for exploratory modeling (VIF = 4.35), the semantic proximity of the survey items confirms substantial latent overlap. Because the present sample size (N=117) was optimized for authentic classroom deployment rather than robust structural equation modeling, running a full structural factor analysis on the current dataset would risk sample-to-variable instability. Consequently, these subscales were retained as separate operational dimensions based on their distinct theoretical orientations (cognitive persistence vs. affective reassurance). Future iterations with larger, multi-institutional cohorts will formally employ Exploratory and Confirmatory Factor Analyses (EFA/CFA) using competitive model comparison (single-factor vs. two-factor models) to evaluate whether these constructs should be empirically collapsed into a unified higher-order affective–motivational construct.

### 7.2. Conclusion and Future Work

This study presented the e-Teacher Assistant, a human-centred and interpretable intelligent educational framework designed to support personalised learning, continuous learner monitoring, and pedagogical decision-making in vocational education. The framework integrates Learning Record Store (LRS) analytics, dynamic learner modelling, fuzzy logic-based assessment, adaptive learning support, and a dual Student Model–Teacher Model architecture within a unified educational environment.

The system was evaluated through a three-month deployment involving 117 vocational education learners and four educators. The findings showed predominantly positive learner perceptions regarding usability, learning support, feedback usefulness, personalisation, autonomous learning, interaction with the AI Assistant, and teacher support. Educators also evaluated the Teacher Model positively, particularly in relation to learner progress monitoring, identification of learning needs, and support for instructional interventions.

The evaluation further showed that the principal learner-experience dimensions were strongly interrelated and that prior familiarity with digital learning tools was associated with more positive evaluations of the system. LRS-based behavioural data complemented the questionnaire findings by documenting learner engagement, repeated examination activity, and the use of AI-assisted support during both theoretical study and examinations.

A central contribution of the proposed framework is its human-in-the-loop orientation. The e-Teacher Assistant is not intended to replace the educator or automate pedagogical decisions. Instead, it provides interpretable learner analytics, adaptive recommendations, and decision-support information while preserving the educator’s responsibility for assessment, instructional planning, learner guidance, and intervention.

Future research should evaluate the framework across a broader range of institutions, subjects, and learner populations. Because the present findings are bounded by self-reported perceptions and observational telemetry, controlled experimental and quasi-experimental studies featuring pre-test/post-test designs and control groups are necessary to determine whether the framework produces causal learning gains. In addition, empirical studies should objectively benchmark teacher decision quality, accuracy, and latency to establish whether human-in-the-loop decision support measurably enhances instructional efficacy beyond self-reported educator satisfaction.

Further technical development should focus on improving learner-model adaptation, refining the explainability of system recommendations, and strengthening the mechanisms used to identify learning difficulties and support timely pedagogical intervention. Future work should also examine how Large Language Models can be incorporated into the framework to generate more personalised learning content, support reflective learning and adaptive practice, and enhance collaboration between educators and intelligent educational systems.

Overall, the findings provide initial empirical support for the feasibility of combining explainable learner modelling, LRS-based learning analytics, adaptive AI assistance, and teacher-centred decision support within an authentic vocational education environment. The results also reinforce the importance of designing educational AI as a collaborative support mechanism that augments, rather than replaces, human pedagogical judgement.

## Figures and Tables

**Table 1:** Comparative Overview of Representative AI-based Educational Assistant Systems and the Proposed e-Teacher Assistant.

System	LRS Integration	XAI Mechanism	Teacher-Facing Analytics	Adaptive Support	Evaluation Type	Sample Size
Sajja et al. [12]	No dedicated LRS reported	No explicit XAI mechanism (LLM/NLP-based)	Yes Limited instructor analytics	Yes (personalised learning support/ pathways)	System design and functional demonstration	No classroom participant sample reported
Mathew et al. [9]	No dedicated LRS reported	None	No dedicated teacher- facing analytics	Limited (NLP-based assistance)	Prototype technical evaluation	~310 questions from 60 Grade-3 students
González et al. [19]	No dedicated LRS (platform activity and interaction logs)	None	Limited/no dedicated teacher- facing analytics	Yes (AI virtual assistant + recommender system)	Multi-cohort course case study/experimental evaluation	n = 311 capstone students
Gutierrez et al. [11]	No dedicated LRS reported	No explicit XAI mechanism	No dedicated teacher- facing analytics	Yes (cognitive + affective adaptation)	2-week controlled experimental evaluation (pre/post + mixed methods)	n = 30 university students
Casalino et al. [29]	No dedicated LRS (VLE/ OULAD data)	Yes (evolving fuzzy classification + IF–THEN explanations)	Limited; no dedicated teacher dashboard	No direct adaptive learner support	Dataset-based evaluation + expert evaluation	OULAD dataset; 21 experts
Ng et al. [23]	No dedicated LRS reported	No explicit XAI mechanism	No dedicated teacher analytics	Yes (personalised SRL prompts, feedback, adaptive responses)	3-week quasi- experimental study	n = 74 students
Papachristou et al. [18]	No dedicated LRS	Yes (Sugeno-type fuzzy inference + interpretable IF–THEN rules)	Yes limited/class-level analytics	Yes (adaptive content/ practice/assessment)	Pilot deployment/experimental evaluation	≈100 learners
Proposed e-Teacher Assistant	Yes (xAPI-style LRS, per-event)	Yes (Sugeno-type fuzzy inference with weighted- average defuzzification and interpretable IF–THEN rules)	Yes (dual Student/Teacher Model, class-level analytics, diagnostic reports)	Yes (content + practice + feedback + progression)	3-month authentic deployment; questionnaire-based evaluation + LRS analytics	N = 117 vocational learners; 4 educators

**Table 2:** Sensitivity Analysis of ELS at Fixed Performance (P=0.55, raw grade 11/20).

Help Profile/Archetype	Questions Helped (Qhelp/10)	Autonomy (A)	Avg Turns (Turns)	Off-Topic Ratio	Uncertainty (U)	Defuzzified ELS	Linguistic Category
1. Fully Autonomous	0/10	1.00 (High)	0.0	0.00	0.00 (Low)	0.628	Average
2. Productive Help-Seeker	2/10	0.80 (High)	1.5	0.00	0.21 (Low)	0.594	Average
3. Hesitant/Verbose Seeker	4/10	0.60 (Medium)	3.5	0.00	0.49 (Medium)	0.492	Average
4. Dependent/Unfocused	7/10	0.30 (Low)	5.0	0.40	0.82 (High)	0.338	Weak

**Table 3:** Internal Consistency Reliability of the Evaluation Dimensions.

Evaluation Dimension	Number of Items	Cronbach’s α
Usability and Ease of Use	3	0.916
Learning Support	>4	0.937
Motivation and Persistence	2	0.873 (Spearman–Brown = 0.876)
Emotional Support and Interaction	4	0.884
Perceived Usefulness, Personalization and Acceptance	4	0.909
Teacher Support and Learning Analytics	3	0.970
Trust, Safety, and Human Oversight	3	0.668

Note: Cronbach’s alpha is not an appropriate reliability estimator for two-item scales; the Spearman–Brown coefficient is therefore also reported for the Motivation and Persistence dimension (inter-item Pearson r = 0.779).

**Table 4:** Spearman’s ρ Correlation Coefficients Between Evaluation Dimensions (N = 117).

Evaluation Dimension	1	2	3	4	5	6	7
Usability and Ease of Use	-						
Learning Support	0.831 ***	-					
Motivation and Persistence	0.706 ***	0.772 ***	-				
Emotional Support and Interaction	0.717 ***	0.725 ***	0.877 ***	-			
Perceived Usefulness, Personalisation and Acceptance	0.782 ***	0.803 ***	0.819 ***	0.788 ***	-		
Teacher Support and Learning Analytics	0.588 ***	0.619 ***	0.675 ***	0.707 ***	0.740 ***	-	
Trust, Safety, and Human Oversight	0.540 ***	0.475 ***	0.534 ***	0.620 ***	0.600 ***	0.541 ***	-

Note: *** *p* < 0.001 (two-tailed).

**Table 5:** Complete Post Hoc Pairwise Comparisons Across Familiarity Groups with 95% Confidence Intervals.

Dimension	Group Medians (IQR)	Frequent vs. Occasional	Frequent vs. Rare/Non-user	Occasional vs. Rare/Non-user
	**Freq** (n=76) | **Occ** (n=3) | **Rare** (n)	U ($p_{\mathrm{exact}}$, r [95% CI])	U ($p_{\mathrm{exact}}$, r [95% CI])	U ($p_{\mathrm{exact}}$, r [95% CI])
**Usability & Ease of Use**	4.67 (0.67) | 4.00 (0.67) | 3.67 (0.67)	612.0 (p<0.001, 0.50 [0.32,0.65])	81.5 (p<0.001, 0.76 [0.57,0.89])	108.0 (p=0.284, 0.25 [−0.17,0.60])
**Learning Support**	4.75 (0.50) | 4.25 (0.50) | 3.75 (0.75)	520.5 (p<0.001, 0.57 [0.40,0.71])	54.0 (p<0.001, 0.84 [0.69,0.94])	86.5 (p=0.083, 0.40 [−0.01,0.70])
**Motivation & Persistence**	4.50 (1.00) | 4.00 (1.00) | 3.50 (1.00)	715.0 (p<0.001, 0.41 [0.21,0.58])	114.0 (p<0.001, 0.67 [0.42,0.83])	118.5 (p=0.439, 0.18 [−0.24,0.54])
**Emotional Support**	4.50 (0.75) | 4.00 (0.75) | 3.75 (1.00)	734.5 (p<0.001, 0.40 [0.20,0.57])	118.5 (p<0.001, 0.65 [0.40,0.82])	120.0 (p=0.463, 0.17 [−0.26,0.53])
**Usefulness & Accept.**	4.75 (0.50) | 4.25 (0.50) | 3.75 (0.50)	598.0 (p<0.001, 0.51 [0.33,0.66])	68.5 (p<0.001, 0.80 [0.62,0.91])	95.5 (p=0.147, 0.34 [−0.08,0.66])
**Teacher Support**	4.67 (0.67) | 4.00 (0.67) | 3.67 (1.00)	798.5 (p=0.002, 0.34 [0.14,0.52])	158.0 (p=0.003, 0.54 [0.24,0.75])	112.5 (p=0.341, 0.22 [−0.20,0.57])
**Trust & Oversight**	4.33 (0.67) | 4.00 (0.67) | 3.33 (0.67)	684.0 (p<0.001, 0.44 [0.24,0.60])	45.0 (p<0.001, 0.87 [0.74,0.95])	45.0 (p=0.001, 0.69 [0.38,0.86])

Note: Bonferroni-adjusted significance threshold is p<0.0167. Exact permutation tests were applied with corrections for ties. r represents the rank-biserial correlation effect size with bootstrap 95% confidence intervals.

**Table 6:** Learning engagement indicators (N=163 learner–chapter records).

Learning Engagement Indicator	Mean ± SD	Median (IQR)	Min–Max
Theory study time (min)	48.21 ± 36.14	33.26 (44.50)	15.17–180.10
Theory sessions	1.91 ± 2.47	1.00 (1.00)	1–25
AI questions	3.17 ± 3.04	3.00 (4.00)	0–27
AI sessions	1.74 ± 2.45	1.00 (2.00)	0–25
Content regenerations	0.56 ± 0.85	0.00 (1.00)	0–4
Study time per topic (min)	10.90 ± 5.24	9.30 (6.85)	4.87–35.01

**Table 7:** Descriptive examination analytics (N=163 learner–chapter records).

Examination Indicator	Mean ± SD	Median (IQR)	Min–Max
Examination attempts	1.50 ± 0.97	1.00 (1.00)	1.00–4.00
First examination grade	15.26 ± 0.96	15.25 (1.25)	11.50–18.10
Final examination grade	16.14 ± 1.57	15.75 (2.00)	13.50–20.00
Best examination grade	16.15 ± 1.58	15.75 (2.00)	13.50–20.00

**Table 8:** Examination AI Support Analytics.

AI Usage Indicator	n	Mean	SD	Median	Min	Max
Help-eligible questions	105	9.40	2.31	11.00	5	11
Questions with help used	105	1.88	1.18	1.00	1	6
Help sessions	105	1.81	1.14	1.00	1	6
Help dialogue turns	105	2.34	1.79	2.00	1	9
Off-topic help turns ^1^	2	1.00	0.00	1.00	1	1
Help-usage ratio	105	0.215	0.149	0.167	0.091	0.833

^1^ The off-topic indicator was computed only for the two learner–chapter records containing at least one off-topic dialogue turn.

## Data Availability

The data presented in this study are available on request from the corresponding author.

## Appendix A. Evaluation Instruments

### Appendix A.1. Learner Evaluation Questionnaire

The learner evaluation questionnaire consisted of 23 five-point Likert-scale items (1 = Strongly Disagree, 5 = Strongly Agree) organised into seven thematic dimensions.

**Construct**	**Code**	**Questionnaire Item**
Usability and Ease of Use	U1	The application was easy to use.
	U2	The instructions were easy to understand.
	U3	Navigation within the application was easy.
Learning Support	LS1	The application helped me better understand the course material.
	LS2	The application helped me identify my mistakes.
	LS3	The feedback provided by the application was useful.
	LS4	The opportunity to repeat activities helped me improve.
Motivation and Persistence	MP1	The system encouraged me to work on my mistakes.
	MP2	I felt comfortable making mistakes without anxiety.
Emotional Support and Interaction	ES1	The system encouraged me to try again.
	ES2	I did not feel punished for making mistakes.
	ES3	I was able to learn from my mistakes.
	ES4	My interaction with the intelligent assistant was useful.
Perceived Usefulness, Personalisation and Acceptance	P1	The application adapted to my learning level and needs.
	P2	The exercises were appropriate for my level of difficulty.
	P3	The application helped me work more independently.
	P4	I would recommend using such an application in other courses.
Teacher Support and Learning Analytics	TS1	The application helped the teacher understand my learning difficulties.
	TS2	The application helped the teacher understand the learning difficulties of the class as a whole.
	TS3	The teacher appeared to adapt the lesson based on the information provided by the application.
Trust, Safety, and Human Oversight	T1	The system provides fair and objective answers.
	T2	The system makes me feel safe while using it.
	T3	I prefer to verify the system’s answers with the teacher.
The learner questionnaire also included two open-ended questions, which were analysed using reflexive thematic analysis:		
Q1. What did you like most about the application?		
Q2. What would you like to improve?		

### Appendix A.2. Teacher Evaluation Questionnaire

The teacher evaluation questionnaire consisted of five five-point Likert-scale items (1 = Strongly Disagree, 5 = Strongly Agree) designed to evaluate educators’ perceptions of the Teacher Model.

**Construct**	**Code**	**Questionnaire Item**
Learning Analytics/Progress Monitoring	TQ1	The learning analytics and reports provided by the Teacher Model helped me monitor learners’ progress more effectively.
Identification of Learning Needs	TQ2	The Teacher Model helped me identify learners’ strengths and learning difficulties.
Instructional Decision Support	TQ3	he class reports and recommendations provided by the Teacher Model were useful for planning appropriate instructional interventions.
Understanding of Instructional Recommendations	TQ4	The information and explanations provided by the Teacher Model helped me understand the proposed instructional recommendations.
Overall Usefulness	TQ5	Overall, I consider the Teacher Model a useful tool for supporting teaching and learner monitoring in vocational education.
The teacher questionnaire also included one open-ended question:		
OQ1. Based on your experience during the classroom deployment, which aspects of the Teacher Model did you find most useful, and what improvements would you recommend for future versions?		
